# Association between Resting Heart Rate and Machine Learning-Based Brain Age in Middle- and Older-Age

**DOI:** 10.14283/jpad.2024.76

**Published:** 2024-04-19

**Authors:** J. Wang, H. Huang, W. Yang, A. Dove, Xiangyu Ma, Weili Xu

**Affiliations:** 1 Department of Epidemiology, College of Preventive Medicine, Third Military Medical University, Gaotanyan Street 30, Shapingba District, Chongqing, 400038 China; 2 Department of Epidemiology and Biostatistics, School of Public Health, Tianjin Medical University, Tianjin, 300070 China; 3 Department of Neurobiology, Care Sciences and Society, Karolinska Institutet, Tomtebodavägen 18A Floor 10, Stockholm, 17165 Sweden

**Keywords:** Resting heart rate, brain age, magnetic resonance imaging, UK Biobank

## Abstract

**Background:**

Resting heart rate (RHR), has been related to increased risk of dementia, but the relationship between RHR and brain age is unclear.

**Objective:**

We aimed to investigate the association of RHR with brain age and brain age gap (BAG, the difference between predicted brain age and chronological age) assessed by multimodal Magnetic Resonance Imaging (MRI) in mid- and old-aged adults.

**Design:**

A longitudinal study from the UK Biobank neuroimaging project where participants underwent brain MRI scans 9+ years after baseline.

**Setting:**

A population-based study.

**Participants:**

A total of 33,381 individuals (mean age 54.74 ± 7.49 years; 53.44% female).

**Measurements:**

Baseline RHR was assessed by blood pressure monitor and categorized as <60, 60–69 (reference), 70–79, or ≥80 beats per minute (bpm). Brain age was predicted using LASSO through 1,079 phenotypes in six MRI modalities (including T1-weighted MRI, T2-FLAIR, T2*, diffusion-MRI, task fMRI, and resting-state fMRI). Data were analyzed using linear regression models.

**Results:**

As a continuous variable, higher RHR was associated with older brain age (β for per 1-SD increase: 0.331, 95% [95% confidence interval, CI]: 0.265, 0.398) and larger BAG (β: 0.263, 95% CI: 0.202, 0.324). As a categorical variable, RHR 70–79 bpm and RHR ≥80 bpm were associated with older brain age (β [95% CI]: 0.361 [0.196, 0.526] / 0.737 [0.517, 0.957]) and larger BAG (0.256 [0.105, 0.407] / 0.638 [0.436, 0.839]), but RHR< 60 bpm with younger brain age (−0.324 [−0.500, −0.147]) and smaller BAG (−0.230 [−0.392, −0.067]), compared to the reference group. These associations between elevated RHR and brain age were similar in both middle-aged (<60) and older (≥60) adults, whereas the association of RHR< 60 bpm with younger brain age and larger BAG was only significant among middle-aged adults. In stratification analysis, the association between RHR ≥80 bpm and older brain age was present in people with and without CVDs, while the relation of RHR 70–79 bpm to brain age present only in people with CVD.

**Conclusion:**

Higher RHR (>80 bpm) is associated with older brain age, even among middle-aged adults, but RHR< 60 bpm is associated with younger brain age. Greater RHR could be an indicator for accelerated brain aging.

**Electronic Supplementary Material:**

Supplementary material is available in the online version of this article at 10.14283/jpad.2024.76.

## Introduction

**R**esting heart rate (RHR) is used to describe the frequency of the cardiac cycle, which is a major determinant of cardiovascular performance and an indicator of autonomic nervous system activity and metabolic rate (1, 2). During the aging process, abnormal changes in RHR may occur due to decreased cardiovascular fitness and autonomic tone or sinus node dysfunction (2–4). An elevated RHR has been linked to an increased risk of mortality and morbidity among older adults (1, 5), as well as faster cognitive decline and a higher risk of dementia (6–11).

Brain magnetic resonance imaging (MRI) is an important tool for understanding brain health, offering an opportunity to evaluate the possible mechanisms underlying brain aging (12, 13). Several studies have investigated the association of elevated RHR with structural or functional brain MRI measures, with inconsistent findings (7, 8, 14–18). Meanwhile, machine learning methods based on multimodal MRI measurements provide the possibility to assess age-related changes in the brain in a more comprehensive manner (19). For example, predicted brain age combines several individual MRI measures (regional brain volume, cortical thickness, fractional anisotropy, functional connection, etc.).

In contrast to individual MRI measures, predicted brain age integrates information on multiple MRI measures into a single metric, factoring in the complex patterns of subtle brain structural changes and the interactions between different brain regions, thus yielding a more sensitive measure of brain aging (20). The difference between predicted brain age and an individual’s actual chronological age, the brain age gap (BAG), is thought to reflect neuroanatomical abnormalities and may be a marker of overall brain health (21). To date, no studies have addressed the association between RHR and brain age in middle-aged and older adults, and it is unclear whether the RHR-brain age relationship varies according to cardiovascular disease (CVD) status.

In the present study, using data from middle-aged (<60) and older (≥60) adults in the UK Biobank, we aimed to: (1) assess the association of RHR with brain age and BAG and (2) explore the role of CVD in these associations.

## Methods

### Study design and population

This study used data from the UK Biobank, a large-scale population-based cohort of 502,412 UK residents aged from 37 to 73 years (22). The UK Biobank study received ethical approval from the National Health Services (NHS) National Research Ethics Service (Ref 11/NW/0382) and all enrolled participants provided informed and written consent.

The baseline survey started in 2006 and a sub-sample of 42,806 participants underwent MRI assessment beginning in 2014. Of them, 34,296 participants with complete brain MRI data were included in the brain age construct. Then, three subsets were defined, including a training set (for training the brain age model, n= 3,484), a validation set (for validating model performance, n= 871), and a testing set (n= 29,941). The training and validation data (in a ratio of 8:2) included people who met health criteria at the time of the scan (n= 4,355), thus excluding people with an ICD-10 diagnosis (field ID 41270), self-reported long-term illness disability or frailty (field ID 2188), diabetes (field ID 2443), history of stroke (field ID 4056), and self-reported fair or poor health status (field ID 2178).

**Figure 1:**
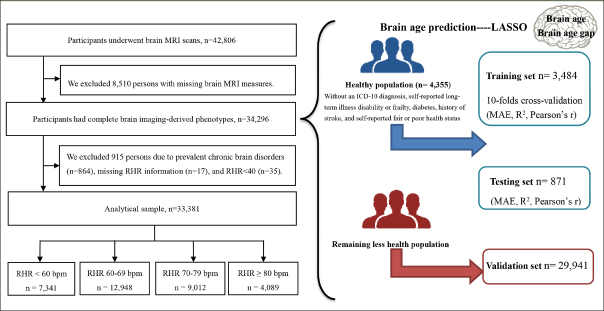
Flowchart of participants included in the study Abbreviation: MRI = magnetic resonance imaging; RHR = resting heart rate; MAE = mean absolute error.

After further excluding 915 participants with prevalent chronic brain disorders (n=864; Table S1), missing information on RHR (n=17), and RHR <40 (n=35), 33,381 participants were included in the RHR-brain aging analysis (Figure 1).

### Data collection

Participants underwent a comprehensive physical examination and clinical evaluation at baseline. In addition, information on demographic characteristics, socioeconomic status, and lifestyle factors was collected through a computerized touchscreen questionnaire (Method S1, Table S2, and Table S3).

### Assessment of RHR

The protocol RHR measurement at the baseline assessment is described in detail in a dedicated document (23). Participants were asked to loosen or remove any restrictive clothing and sit with their feet parallel to each other, toes pointing forward, and soles of the feet resting flat on the floor. RHR was then measured on the participant’s left arm (or right arm if the left side was affected by amputation, shunt, mastectomy, or axillary clearance) with the Omron 705 IT electronic blood pressure monitor (OMRON Healthcare Europe B.V. Kruisweg 577 2132 NA Hoofddorp). Two readings were recorded for each participant and the average of the two measurements was used in our analysis. When considered as a categorical variable, RHR was classified as <60, 60–70 (reference), 70–79, and ≥80 beats per minute (bpm) according to previous studies (6, 10).

### MRI data acquisition and pre-processing

Details on the UK Biobank brain MRI data acquisition and processing are available on the UK Biobank website in the brain scan protocol and brain imaging documentation (Method S2) (24, 25). Summary measures of imaging-derived phenotypes were generated with an image-processing pipeline developed and run on behalf of the UK Biobank, using publicly available image processing tools FSL (the FMRIB Software Library, version 5.0.10, http://fsl.fmrib.ox.ac.uk/fsl) and FreeSurfer (version 6.0) (26). Finally, 1,079 neuroimaging phenotypes were generated from five modalities, including T1-weighted (n=165), T2-FLAIR (n=1; total volume of white-matter hyperintensities), SWI (n=14), diffusion-MRI (n=675), task fMRI (n=14), and resting-state fMRI (n=210) (Table S4). All MRI phenotypes were Z-transformed before analysis.

### Statistical analysis

RHR was operationalized as both a continuous and a categorical variable. Baseline characteristics of the study population by RHR category were analyzed using chi-square tests for categorical variables and one-way ANOVA for continuous variables. Alpha was set at 0.05 for all analyses and results were corrected for multiple comparisons using the false discovery rate (FDR). All statistical analyses were performed using Python (version 3.8.0 with sklearn), Stata SE 16.0 (Stata Corp, College Station, Texas), and R (version 4.1.1).

### Assessment of brain age and BAG

Least Absolute Shrinkage and Selection Operator (LASSO) regression was trained in the training dataset. The performance of the model was evaluated using mean absolute error (MAE) on a standardized validation dataset (with mean absolute error =3.232, R2 = 0.690, and Pearson’s r = 0.830). Next, the model was used to predict brain age in the entire population (the phenotypes and the corresponding coefficients are list in Table S5). Moreover, we corrected the age bias in brain age prediction as the corrected brain age=(original brain age-β)/α (where coefficients a and β are the slope and intercept of the linear regression model used to estimate the brain age in the training set: brain age_(training set)=α*chronological age_(training set)+β) (27). BAG was defined as corrected brain age minus chronological age. A positive BAG means that the predicted brain age is higher than chronological age (i.e., accelerated aging); a negative BAG means that the predicted brain age is lower (i.e., delayed aging) (21).

### Association between RHR and Brain age

Linear regression models were used to estimate the β-coefficients and 95% confidence intervals (CIs) for the association between RHR and brain age or BAG among middle-aged (<60 year) and older (≥60 years) participants. The basic models were adjusted for age, sex, and education. Multivariable-adjusted models were further adjusted for race, socioeconomic status, BMI, alcohol consumption, smoking, physical activity, social contact, hypertension, diabetes, heart disease, medications that reduce RHR (beta blockers and calcium blockers), and apolipoprotein E (APOE) ε4. Stratified analyses were performed to explore the role of CVD in the association of RHR with brain age and BAG. Statistical interaction was examined by creating an indicator variable with the cross-product of two variables.

In sensitivity analysis, we repeated the analyses after 1) stratifying by APOE ε4, physical activity, and polygenic risk score for AD (PRSAD); 2) further adjusting for DASH diet score and PRSAD; and 3) performing multiple imputation for missing values of covariates using Fully Conditional Specification (FCS) method.

## Results

### Baseline characteristics

The mean age at baseline was 54.74 ± 7.49, and 53.44% of participants were female. Of the participants, the mean RHR was 67.79 ± 10.43 bpm. Compared to participants with lower RHR, those with higher RHR were more likely to be older and female; to have, BMI and social contact, lower education level, socioeconomic status (SES), and physical activity; and to be non-drinkers and current smokers. In addition, they tended to have a history of hypertension, diabetes, and heart disease, and higher usage of calcium channel blockers but lower usage of beta-blockers (Table 1).

**Table 1:** Baseline characteristics of the study populations by resting heart rate (RHR) group (n= 33,381)

**Characteristics**	**RHR (bpm)**				**P value**
	**<60 (n = 7,341, 22.0%)**	**60–69 (n = 12,948, 38.8%)**	**70–79 (n = 9,012, 27.0%)**	**≥ 80** **(n = 4,089, 12.2%)**	
Age (y)	55.06 (7.45)	54.74 (7.44)	54.54 (7.58)	54.78 (7.54)	<0.001
Female	2,996 (40.81)	7,137 (55.12)	5,368 (59.57)	2,321 (56.89)	<0.001
Race-White	6,821 (93.16)	11,965 (92.63)	8,326 (92.64)	3,774 (92.80)	0.526
Townsend deprivation index	−2.72 (−3.95, −0.75)	−2.63 (−3.90, −0.57)	−2.55 (−3.85, −0.38)	−2.47 (−3.79, −0.14)	<0.001
Education (college)	3,506 (48.43)	5,942 (46.60)	4,082 (46.03)	1,818 (45.47)	0.005
BMI (kg/m^2^)	25.77 (3.60)	26.22 (3.93)	26.83 (4.33)	27.82 (4.95)	<0.001
*Alcohol consumption status*					<0.001
Never	117 (1.59)	284 (2.19)	248 (2.75)	132 (3.24)	
Former drinker	132 (1.80)	253 (1.95)	170 (1.89)	111 (2.72)	
Current drinker	7,088 (96.61)	12,405 (95.85)	8,591 (95.36)	3,834 (94.04)	
*Smoking status*					<0.001
Never	4,377 (59.76)	7,879 (60.99)	5,567 (61.91)	2,538 (62.30)	
Former smoker	2,541 (34.69)	4,333 (33.54)	2,849 (31.68)	1,203 (29.53)	
Current smoker	406 (5.54)	706 (5.47)	576 (6.41)	333 (8.17)	
Regular physical activity	4,563 (62.95)	7,771 (60.93)	5,258 (59.30)	2,366 (59.25)	<0.001
Regular social contact	2686 (37.05)	4983 (39.07)	3609 (40.70)	1627 (40.75)	<0.001
Hypertension	3,101 (42.24)	5,267 (40.68)	4,094 (45.43)	2,333 (57.18)	<0.001
Diabetes	134 (1.83)	271 (2.09)	244 (2.71)	225 (5.51)	<0.001
Heart disease	486 (6.62)	367 (2.83)	236 (2.62)	126 (3.09)	<0.001
*Medication*					
Beta-blockers	673 (9.17)	366 (2.83)	146 (1.62)	44 (3.58)	<0.001
Calcium blockers	205 (2.79)	396 (3.06)	318 (3.53)	205 (5.02)	<0.001
APOE e4 carrier	1,769 (28.69)	2,972 (27.31)	2,057 (27.32)	900 (26.97)	0.165
Brain age	63.36 (6.13)	63.36 (6.06)	63.49 (6.31)	64.09 (6.35)	<0.001
Brain age gap	0.01 (4.90)	0.28 (4.94)	0.62 (5.32)	1.28 (5.30)	<0.001

Abbreviations: APOE ε4 = apolipoprotein E epsilon; BMI = body mass index; PRSAD = polygenetic risk score of Alzheimer’s disease. Missing data: Race = 88; Education = 524; BMI = 30; Townsend deprivation index = 31; Alcohol consumption = 16; Smoking = 73; Regular social contact = 518; Regular physical activity = 1,101; APOE ε4 carrier = 5468.

### Association of resting heart rate with brain age and BAG

Among all participants, the time interval between study entry and MRI assessment was 8.94 ± 1.78 years. Higher RHR was associated with older brain age (β for per 1-SD increase: 0.331, 95% CI: 0.265, 0.398) and larger BAG (β: 0.263, 95% CI: 0.202, 0.324) when RHR was analyzed as a continuous variable (Table 2). Furthermore, when RHR was categorized, RHR 70–79 bpm and RHR ≥80 bpm were related to older brain age (β [95% CI]: 0.361 [0.196, 0.526] / 0.737 [0.517, 0.957]) and larger BAG (β [95% CI]: 0.256 [0.105, 0.407] / 0.638 [0.436, 0.839]) compared to RHR 60–69 bpm (Table 2). In addition, RHR <60 bpm was associated with younger brain age (β: −0.324, 95% CI: −0.500, −0.147) and smaller BAG (β: −0.230, 95% CI: −0.392, −0.067). The results of basic-adjusted models were similar to the multi-adjusted models (Table 2). Regression coefficients for the covariates in relation to brain volumes were shown in Supplementary Table 7.

**Table 2:** The association of resting heart rate (RHR) with brain age and brain age gap (BAG)

**RHR**	**Brain age**		**BAG**	
	β (95% CI) *	**β (95% CI) †**	**β (95% CI) †**	**β (95% CI) †**
All participants (N = 33,381)				
Continuous (per 1-SD increase) Categories	0.432 (0.375, 0.489) ‡	0.331 (0.265, 0.398) ™	0.399 (0.346, 0.452) ‡	0.263 (0.202, 0.324) †
< 60 bpm	−0.355 (−0.515, −0.196) ‡	−0.324 (−0.500, −0.147) ‡	−0.311 (−0.458, −0.164) ‡	−0.230 (−0.392, −0.067) †
60–69 bpm	Reference	Reference	Reference	Reference
70–79 bpm	0.411 (0.262, 0.561) ‡	0.361 (0.196, 0.526) ‡	0.346 (0.209, 0.484) ‡	0.256 (0.105, 0.407) †
≥ 80 bpm	1.069 (0.873, 1.265) ‡	0.737 (0.517, 0.957) ‡	1.033 (0.853, 1.213) ‡	0.638 (0.436, 0.839) †
Middle-aged (40–60 years, n = 22,923)				
Continuous (per 1-SD increase) Categories	0.412 (0.345, 0.480) ‡	0.271 (0.194, 0.348) ‡	0.386 (0.324, 0.449) ‡	0.201 (0.130, 0.272) †
< 60 bpm	−0.405 (−0.592, −0.218) ‡	−0.270 (−0.475, −0.066) ‡	−0.388 (−0.561, −0.216) ‡	−0.196 (−0.384, −0.007)
60–69 bpm	Reference	Reference	Reference	Reference
70–79 bpm	0.361 (0.189, 0.534) ‡	0.322 (0.133, 0.511) ‡	0.307 (0.147, 0.467) ‡	0.222 (0.048, 0.396) †
≥ 80 bpm	0.973 (0.745, 1.201) ‡	0.604 (0.349, 0.858) ‡	0.920 (0.709, 1.131) ‡	0.461 (0.226, 0.695) †
Older-aged (60+ years, n = 10,458)				
Continuous (per 1-SD increase) Categories	0.457 (0.349, 0.566) ‡	0.438 (0.310, 0.565) ‡	0.411 (0.312, 0.510) ‡	0.375 (0.258, 0.491) †
< 60 bpm	−0.255 (−0.558, 0.048)	−0.431 (−0.774, −0.089)	−0.153 (−0.429, 0.123)	−0.298 (−0.610, 0.014)
60–69 bpm	Reference	Reference	Reference	Reference
70–79 bpm	0.485 (0.195, 0.774) ‡	0.399 (0.074, 0.724) ‡	0.398 (0.133, 0.662) ‡	0.281 (-0.014, 0.576)
≥ 80 bpm	1.239 (0.866, 1.612) ‡	0.974 (0.549, 1.400) ‡	1.237 (0.898, 1.577) ‡	0.972 (0.585, 1.359) †
P-interaction	<0.001†		0.031 †	

* Model adjusted for age, sex, and education. † Model adjusted for age, sex, education, race, Socioeconomic status, body mass index, alcohol consumption, smoking, physical activity, social contact, hypertension, diabetes, heart disease, beta-blockers, calcium blockers, and APOE ε4. *1* FDR P <0.05.

In age-stratified analyses, the association of RHR ≥80 bpm with older brain age and larger BAG was strongest among middle-aged participants, but was also present among older participants (Table 2). In contrast, the association between RHR <60 bpm and younger brain age and smaller BAG was only significant among middle-aged participants (Table 2). There was a significant multiplicative interaction between RHR and age group for brain age and BAG (P-interaction= 0.001 and 0.031).

### Role of CVD

**Figure 2:**
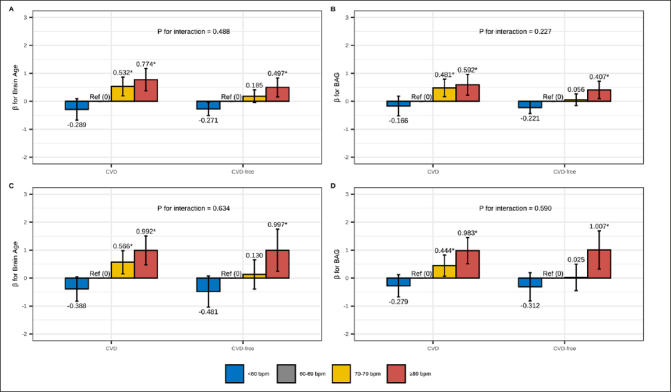
Association of RHR with brain age and BAG: stratified by CVD The figure represents the association between RHR (reference: 60–69 bpm) and brain age and BAG among CVD and CVD-free participants: (A/B) RHR-brain age/BAG association among middle-aged adults; (C/D) RHR-brain age/BAG association among older-aged adults. Model adjusted for age, sex, education, race, Socioeconomic status, body mass index, alcohol consumption, smoking, physical activity, social contact, diabetes, beta-blockers, calcium blockers, and APOE ε4. The height of each bar in the plot represents the point estimate of β, while the error bars represent the 95% confidence intervals of β. * FDR P< 0.05.

In CVD-stratified analyses (Figure 2 and Table S6), RHR ≥80 bpm was associated with older brain age and larger BAG compared to RHR 60–69 bpm among both CVD and CVD-free participants, regardless of age group. However, the association between RHR 70–79 bpm and older brain age was only present in participants with CVD. There was no statistical interaction between RHR and CVD on brain age or BAG in the multi-variable adjusted models (all P-interactions>0.05).

### Supplementary analysis

Largely similar results were obtained after we conducted the following sensitivity analyses: 1) stratifying by APOE ε4 (Table S8), physical activity level (Table S9), and PRSAD (Table S10); 2) further adjusting for DASH diet score and PRSAD (Table S11 and S12); and 3) performing multiple imputation for missing values of covariates (Table S13).

## Discussion

In this large neuroimaging study with multimodal MRI measures, we constructed a brain age prediction model based on LASSO regression and found that 1) RHR 70–79 bpm and RHR≥80 bpm was associated with older brain age and larger BAG among both middle-aged and older adults; 2) RHR <60 bpm was associated with younger brain age and smaller BAG only among middle-aged participants; and 3) the association of RHR 70–79 with older brain age was only present among people with CVDs.

### RHR and brain aging

Several previous studies have linked RHR to individual MRI measures (7, 8, 14–18). Some studies have reported associations between elevated RHR and more severe brain lesions including white matter hyperintensity (8, 14, 16), brain infarction (16), and ischemic lesions (8), while others related higher RHR to smaller brain volumes including total brain (17) and some subfields of the hippocampus (7), but with some inconsistent findings (8). Limited studies evaluated the association between elevated RHR and poor brain functional connectivity (15, 18). However, these studies offer limited support for the association between RHR and brain aging as they solely examined separate MRI metrics that may not adequately capture the extent of brain aging in individuals. By ‘learning’ the correspondence between patterns in structural or functional multimodal neuroimaging data and an age ‘label’, machine-learning algorithms can formulate massively high-dimensional regression models, fitting large neuroimaging datasets as independent variables to predict chronological age as the dependent variable. Predicted brain age and derived BAG provide a more comprehensive description of brain aging (20). In the present study, we found that elevated RHR was associated with unhealthy brain aging, including older brain age and larger BAG. Our findings provide further evidence that elevated RHR may play a role in the process of brain aging. Further, after stratification by age, the association of elevated RHR with older brain age and larger BAG remains significant among middle-aged and older adults. These results add a growing body of evidence that the effect of RHR on brain aging may begin as early as middle age. To our knowledge, this is the first study to address the association of RHR with brain age and BAG, supported by multimodal brain MRI data.

### Role of CVD in RHR-brain age association

RHR is a strong predictor of cardiovascular events including hypertension, myocardial infarction, angina, congestive heart failure, and atrial fibrillation (28, 29). Moreover, these non-stroke CVDs are associated with brain aging (30–32). However, to date, the comprehensive differences in the RHR-brain aging association across different CVD states have not been well known. In the current study, we found that the association between RHR ≥80 bpm and brain aging was present not only in the CVD population but also in the non-CVD population, with no significant interaction between RHR and CVD on brain aging. Our findings suggest that monitoring RHR is beneficial for indicating brain aging among individuals both with and without CVD.

### Potential mechanisms underlying RHR-brain age association

There are several mechanisms whereby elevated RHR may link to brain aging. First, increased RHR may lead to decreased cerebral blood flow, aggravating ischemia or white matter lesions, and lacunar infarction in some brain regions (33, 34). In addition, high RHR can lead to cerebral hypoxia, which causes vascular endothelial dysfunction and enhanced coagulation activity, bringing vascular and neurodegenerative damage in the brain (35, 36). Second, elevated RHR may also be a marker of autonomic dysfunction, which may drive the development of cognitive decline and dementia by activating inflammatory pathways and increasing the levels of inflammatory markers such as hs-CRP, IL-6, and fibrinogen (37, 38). Third, brain aging and CVDs may share similar pathogenic processes (39, 40). However, the association of elevated RHR with older brain age and larger BAG remained significant among CVD-free people and after controlling for traditional cardiovascular risk factors, which may indicate an independent effect of RHR on brain aging. Future studies are warranted to better understand the mechanisms underlying the influence of RHR on brain health.

### Strength and limitations

Strengths of this study include the large-scale community-based design with a comprehensive data collection procedure. Additionally, the UK Biobank provides image-derived phenotypes of various brain measures, offering an opportunity to calculate multimodal brain age using machine learning models. Nonetheless, some limitations should be acknowledged. First, the UK Biobank participants were volunteers and generally healthier and more highly educated than the general population (41). We noted that all participants who underwent MRI scans had high economic status, and the differences in SES between the different RHR groups may be due to the large sample. Moreover, our analytical sample consisted of participants who underwent brain MRI scans and were free from chronic brain disorders, which is a relatively healthier subset in the overall UK Biobank population. This might have contributed to a misestimating of the magnitude of the association between elevated RHR and brain aging. Furthermore, our findings may only be generalizable to demographically similar cohorts, and this limitation precludes the generalization of these findings to the general population. Second, although the direction of the associations of RHR with brain age and BAG may indicate the potential effect of RHR itself on brain aging, these results cannot draw causal inferences. However, a subgroup of approximately 10,000 participants from UKB is currently undergoing repeat brain MRI scans (42), providing an opportunity for future studies to explore the longitudinal association between RHR and brain aging. Third, selection bias might have occurred due to missing data. However, the results were not much altered after repeating the analysis using multiple imputations for missing variables.

## Conclusion

In conclusion, our study provides new evidence that elevated RHR may associated with brain aging across middle- and older-age and decreased RHR (<60 bpm) was related to younger brain age among middle-aged adults. CVD may play a role in the association of RHR 70–79 bpm with older brain age and larger BAG. The RHR is a very commonly used parameter in clinical settings and can be obtained more easily and quickly than complex cognitive assessments. Moreover, there are a number of drugs (such as beta-blockers) that can be used to control RHR, and if a longitudinal or causal association between RHR and brain aging can be identified, it may open up new possibilities for intervention and treatment of cognitive impairment and dementia. Thus, future longitudinal studies or Mendelian randomization are needed to explore the longitudinal effects of RHR on brain aging.

## Data Availability

*Data Availability:* The datasets generated and analysed during the current study are available in the UK Biobank repository, http://www.ukbiobank.ac.uk.

## Supplementary methods

Supplementary material, approximately 28 KB.

Supplementary material, approximately 152 KB.

## Notes

*Ethical Standards:* The authors assert that all procedures contributing to this work comply with the ethical standards of the relevant national and institutional committees on human experimentation and with the Helsinki Declaration of 1975, as revised in 2008.
