# Supplementary material for: Association between Resting Heart Rate and Machine Learning-Based Brain Age in Middle- and Older-Age
Source: J Prev Alzheimers Dis. 2024 Apr 19;11(4):1140–7. doi: 10.14283/jpad.2024.76 (PMC11266275; doi:10.14283/jpad.2024.76)
Supplement: Supplementary file 1 — Supplementary material, approximately 28 KB. [file mmc1.docx]

**Supplementary methods**

**Supplementary Method 1. Assessment of covariates**

Ethnic background was dichotomized as white or non-white. Socioeconomic status (SES) was ascertained by the Townsend Deprivation Index (TDI), an indicator of socioeconomic deprivation based on neighborhood levels of unemployment, household overcrowding, car non-ownership, and home non-ownership ^(1)^. Education level was categorized as college or non-college. Body mass index (BMI) was calculated as weight (kg) / height (m^2^). Smoking and drinking status were categorized as never, former, or current smoker/drinker. Regular physical activity was defined as at least 150 minutes of moderate activity per week, 75 minutes of vigorous activity per week, or an equivalent combination ^(2)^. Social contact was evaluated based on responses to the question "How often do you visit friends or family or have them visit you?": Almost daily, 2-4 times a week, about once a week, about once a month, once every few months, never or almost never, no friends/family outside household, or do not know/prefer not to answer (treated as missing). These responses were further dichotomized as high or low according to the median.

Blood pressure was measured using the Omron 705 IT electronic blood pressure monitor at baseline. Two readings were recorded for each participant and the average of the two measurements was calculated. Hypertension was defined as systolic blood pressure ≥140 mm Hg, diastolic blood pressure ≥90 mm Hg, use of antihypertensive drugs, or a history of hypertension. Heart disease including myocardial infarction, angina, congestive heart failure, and atrial fibrillation was assessed based on self-reported and registered medical history. Diabetes was defined as hemoglobin A1c ≥6.5%, fasting plasma glucose ≥126 mg/dl, having a history of diabetes, or the use of glucose-lowering medications ^(3)^. CVD was defined as the presence of hypertension or heart disease ^(4)^. Use of medications – including those that reduce RHR such as beta-blockers (BB, C07) and calcium channel blockers (CCB; C08, C07FB, C09BB, and C09DB) – was determined using Anatomical Therapeutic Chemical (ATC) codes (<https://www.genome.jp/kegg-bin/get_htext?br08303>). Adherence to the dietary approaches to stop hypertension (DASH) eating pattern was calculated based on the intake level of 8 food groups: fruit, vegetables, whole grains, nuts and legumes, low-fat dairy products, sodium, red and processed meat, and sweetened beverages ^(5, 6)^ (**Supplementary Table 3**). Moreover, the *APOE* gene was genotyped and dichotomized as carriers vs. non-carriers of the ε4 allele. Alzheimer's disease (AD)-related polygenic risk score (PRS_AD_) was obtained from UKB’s Standard PRS Set (field ID: 26206), which was calculated based on meta-analysis of external genome-wide association studies ^(7)^.

**Supplementary Method 2. MRI data acquisition and pre-processing**

Participants were scanned with a Siemens Skyra 3T scanner with a standard Siemens 32-channel head coil. T1-weighted imaging (Resolution: 1.0 × 1.0 × 1.0 mm; field-of-view: 208 × 256 × 256 matrix), T2 FLAIR imaging (Resolution: 1.05 × 1.0 × 1.0 mm; field-of-view: 192 × 256 × 256 matrix), susceptibility-weighted structural imaging (SWI, Resolution: 0.8 × 0.8 × 3 mm; field-of-view: 256 × 288 × 48 matrix; 2 echo TEs=9.42, 20 ms), and task and resting-state fMRI (Resolution: 2.4 × 2.4 × 2.4 mm; field-of-view: 88 × 88 × 64 matrix; TR: 0.735 s; TE: 39ms; GE-EPI with × 8 multi-slice acceleration) were performed. Diffusion imaging was applied to estimate the diffusion properties reflecting the integrity of microstructural tissue compartments, and 36 slices (resolution: 2.0 × 2.0 × 2.0 mm; field-of-view:104 × 104 × 72 matrix) were obtained using an echo plane, single-shot Stejskal-Tanner pulse sequence (echo time: 92 ms) in 50 distinct diffusion-weighted directions (b= 1000 and 2000 sec/mm^2^).

**Reference**

1. P T. Deprivation. *Journal of Social Policy*. 1987;16(2): 125-46. 10.1017/S0047279400020341.

2. Lloyd-Jones DM, Hong Y, Labarthe D, et al. Defining and setting national goals for cardiovascular health promotion and disease reduction: the American Heart Association's strategic Impact Goal through 2020 and beyond. *Circulation*. 2010;121(4):586-613. 10.1161/circulationaha.109.192703.

3. Association AD. 2. Classification and Diagnosis of Diabetes: Standards of Medical Care in Diabetes-2018. *Diabetes Care*. 2018;41(Suppl 1):S13-s27. 10.2337/dc18-S002.

4. Biddinger KJ, Emdin CA, Haas ME, et al. Association of Habitual Alcohol Intake With Risk of Cardiovascular Disease. *JAMA Netw Open*. 2022;5(3):e223849. 10.1001/jamanetworkopen.2022.3849.

5. Fung TT, Chiuve SE, McCullough ML, Rexrode KM, Logroscino G, Hu FB. Adherence to a DASH-style diet and risk of coronary heart disease and stroke in women. *Archives of Internal Medicine*. 2008;168(7):713-20. 10.1001/archinte.168.7.713.

6. Mompeo O, Freidin MB, Gibson R, et al. Genome-Wide Association Analysis of Over 170,000 Individuals from the UK Biobank Identifies Seven Loci Associated with Dietary Approaches to Stop Hypertension (DASH) Diet. *Nutrients*. 2022;14(20):4431. 10.3390/nu14204431.

7. Thompson DJ, Wells D, Selzam S, et al. UK Biobank release and systematic evaluation of optimised polygenic risk scores for 53 diseases and quantitative traits. 2022:2022.06.16.22276246. 10.1101/2022.06.16.22276246 %J medRxiv.
