# Supplementary material for: Association between Resting Heart Rate and Machine Learning-Based Brain Age in Middle- and Older-Age
Source: J Prev Alzheimers Dis. 2024 Apr 19;11(4):1140–7. doi: 10.14283/jpad.2024.76 (PMC11266275; doi:10.14283/jpad.2024.76)
Supplement: Supplementary file 2 — Supplementary material, approximately 152 KB. [file mmc2.docx]

**Supplementary**

[Supplementary Table 1. Self-reported health variables codes used for exclusion 2](#_Toc147495921)

[Supplementary Table 2. UK Biobank Field codes for all covariates 3](#_Toc147495922)

[Supplementary Table 3 DASH dietary pattern and corresponding Field ID. 4](#_Toc147495923)

[Supplementary Table 4 Missing number for brain imaging-derived phenotypes 7](#_Toc147495924)

[Supplementary Table 5. The coefficient of each phenotype in LASSO 21](#_Toc147495925)

[Supplementary Table 6. The RHR-brain age association: stratified by CVD 28](#_Toc147495926)

[Supplementary Table 7. The RHR-brain age association: stratified by *APOE* ε4 29](#_Toc147495927)

[Supplementary Table 8. The RHR-brain age association: stratified by physical activity 30](#_Toc147495928)

[Supplementary Table 9. The RHR-brain age association: stratified by PRS_AD_ 31](#_Toc147495929)

[Supplementary Table 10. The RHR-brain age association: further adjusted for DASH 32](#_Toc147495930)

[Supplementary Table 11. The RHR-brain age association: further adjusted for PRS_AD_. 33](#_Toc147495931)

[Supplementary Table 12. The RHR-brain age association: after multiple imputation 34](#_Toc147495932)

# Supplementary Table 1. Self-reported health variables codes used for exclusion criteria on initial population.

| **Self-reported illness** | **Code (Field ID 20002 and 20003)** |
| --- | --- |
| Dementia or Alzheimer’s disease | 1263 |
| Parkinson’s disease | 1262 |
| Chronic degenerative neurological | 1258 |
| Guillain-Barré syndrome | 1256 |
| Multiple Sclerosis | 1261 |
| Other demyelinating disease | 1397 |
| Stroke or ischemic stroke | 1081 |
| Brain cancer | 1032 |
| Brain hemorrhage | 1491 |
| Brain/intracranial abscess | 1245 |
| Cerebral aneurysm | 1425 |
| Cerebral palsy | 1433 |
| Encephalitis | 1246 |
| Epilepsy | 1264 |
| Head injury | 1266 |
| Infections of the nervous system | 1244 |
| Ischemic stroke | 1583 |
| Meningeal cancer | 1031 |
| Meningioma (benign) | 1659 |
| Meningitis | 1247 |
| Motor Neuron Disease | 1259 |
| Neurological injury/trauma | 1240 |
| Spina bifida | 1524 |
| Subdural hematoma | 1083 |
| Subarachnoid hemorrhage | 1086 |
| Transient ischemic attack | 1082 |

# Supplementary Table 2. UK Biobank Field codes for all covariates used in the current study.

| **Variable** | **Code** |
| --- | --- |
| Age | 21300 |
| Sex | 31 |
| Education | 6138 |
| BMI | 21001 |
| Race | 21000 |
| Smoking Status | 20116 |
| Alcohol consumption status | 20117 |
| Physical activity | 22032; 894; 914; 884; 904 |
| Social contact | 6160 |
| Hypertension | Field ID 20002: 1065, 1072; 6177, 6153 (medication); 2966; 6150; 4080 (Systolic blood pressure); 4079 (Diastolic blood pressure); 131286 (ICD) |
| Diabetes | Field ID 20002: 1220, 1222, 1223; 2443; 2976; 30750 (HbA1c); 30740 (Glucose); 6153, 6157 (medication); 130706 (ICD) |
| Heart disease | Field ID 20002: 1074, 1075; 6150; 3627, 131297 (ICD, angina); 131350 (ICD, atrial fibrillation); 3894, 131354 (ICD, heart failure) |
| Medication | 20003 |
| *APOE* ε4 | rs428358; rs7412 |
| PRS_AD_ | 26206 |

# Supplementary Table 3 The 8 food groups of dietary approaches to stop hypertension (DASH) dietary pattern and corresponding UK Biobank Field ID.

| **Food group** | **Field ID** | **Description** |
| --- | --- | --- |
| **Fruit** | 104410 | Stewed fruit intake |
|  | 104420 | Prune intake |
|  | 104430 | Dried fruit intake |
|  | 104440 | Mixed fruit intake |
|  | 104450 | Apple intake |
|  | 104460 | Banana intake |
|  | 104470 | Berry intake |
|  | 104480 | Cherry intake |
|  | 104490 | Grapefruit intake |
|  | 104500 | Grape intake |
|  | 104510 | Mango intake |
|  | 104520 | Melon intake |
|  | 104530 | Orange intake |
|  | 104540 | Satsuma intake |
|  | 104550 | Peach/nectarine intake |
|  | 104560 | Pear intake |
|  | 104570 | Pineapple intake |
|  | 104580 | Plum intake |
|  | 104590 | Other fruit intake |
|  | 100190 | Orange juice intake |
|  | 100200 | Grapefruit juice intake |
|  | 100210 | Pure fruit/vegetable juice intake |
| **Vegetables** | 104060 | Mixed vegetable intake |
|  | 104070 | Vegetable pieces intake |
|  | 104080 | Coleslaw intake |
|  | 104090 | Side salad intake |
|  | 104100 | Avocado intake |
|  | 104110 | Broad bean intake |
|  | 104120 | Green bean intake |
|  | 104130 | Beetroot intake |
|  | 104140 | Broccoli intake |
|  | 104150 | Butternut squash intake |
|  | 104160 | Cabbage/kale intake |
|  | 104170 | Carrot intake |
|  | 104180 | Cauliflower intake |
|  | 104190 | Celery intake |
|  | 104200 | Courgette intake |
|  | 104210 | Cucumber intake |
|  | 104220 | Garlic intake |
|  | 104230 | Leek intake |
|  | 104240 | Lettuce intake |
|  | 104250 | Mushroom intake |
|  | 104260 | Onion intake |
|  | 104270 | Parsnip intake |
|  | 104280 | Pea intake |
|  | 104290 | Sweet pepper intake |
|  | 104300 | Spinach intake |
|  | 104310 | Sprouts intake |
|  | 104320 | Sweetcorn intake |
|  | 104330 | Sweet potato intake |
|  | 104340 | Fresh tomato intake |
|  | 104350 | Tinned tomato intake |
|  | 104360 | Turnip/swede intake |
|  | 104370 | Watercress intake |
|  | 104380 | Other vegetables intake |
| **Whole grains** | 100950&20091 | Wholemeal sliced bread intake |
|  | 100950&20091 | Seeded sliced bread intake |
|  | 100950&20091 | Mixed sliced bread intake |
|  | 100950&20091 | Other sliced bread.intake |
|  | 101020&20092 | Wholemeal baguette intake |
|  | 101020&20092 | Seeded baguette intake |
|  | 101020&20092 | Mixed baguette intake |
|  | 101020&20092 | Other baguette intake |
|  | 101160&20094 | Wholemeal bread roll intake |
|  | 101160&20094 | Seeded bread roll intake |
|  | 101160&20094 | Mixed bread roll intake |
|  | 101160&20094 | Other bread roll intake |
|  | 101090&20093 | Wholemeal bap intake |
|  | 101090&20093 | Seeded bap intake |
|  | 101090&20093 | Mixed bap intake |
|  | 101090&20093 | Other bap intake |
|  | 101250 | Crispbread intake |
|  | 101260 | Oatcakes intake |
|  | 101270 | Other bread intake |
|  | 100770 | Porridge intake |
|  | 100800 | Muesli intake |
|  | 100810 | Oat crunch intake |
|  | 100840 | Bran cereal intake |
|  | 100850 | Whole-wheat cereal intake |
|  | 102720 | Wholemeal pasta intake |
|  | 102740 | Brown rice intake |
|  | 102780 | Other grain intake |
| **Nuts and legumes** | 104000 | Baked bean intake |
|  | 104010 | Pulses intake |
|  | 20088 | Hummus spread sauces intake |
|  | 20088 | Peanut butter spread sauces intake |
|  | 103270 | Tofu intake |
|  | 103280 | Quorn intake |
|  | 103260 | Vegetarian sausages/burgers intake |
|  | 103290 | Other vegetarian alternative intake |
|  | 102450 | Seeds intake |
|  | 102430 | Salted nuts intake |
|  | 102410 | Salted peanuts intake |
|  | 102440 | Unsalted nuts intake |
|  | 102420 | Unsalted peanuts intake |
| **Low-fat dairy** | 102850 | Low fat cheese spread intake |
|  | 102810 | Low fat hard cheese |
|  | 102870 | Cottage cheese intake |
|  | 100520&100920 | Skimmed milk intake |
|  | 100520&100920 | Semi-skimmed milk intake |
|  | 102090&20106 | Low fat yogurt consumers, yogurt intake |
| **Sodium** | 30510, 30520, 30530, 21001& 21022 | Sodium using the predictive equation of the INTERSALT study based on casual urinary sodium, potassium, and creatinine concentrations. Males: sodium intake [mg]=(25.46+0.46*casual urinary sodium [mmol/L]-2.75* creatinine [mmol/L]-0.13*potassium [mmol/L]+4.10*BMI [kg/m^2^]+0.26 Age [years]+17.05)*23; Females: sodium intake [mg]=(5.07+0.34*casual urinary sodium [mmol/L]-2.16*creatinine [mmol/L]-0.09*potassium [mmol/L]+2.39*BMI [kg/m^2^]+2.35*Age [years]-0.03*Age^2^ [years^2^]+12.82)*23 |
| **Red and processed meat** | 103010 | Sausage intake |
|  | 103020 | Beef intake |
|  | 103030 | Pork intake |
|  | 103040 | Lamb intake |
|  | 103060 | Poultry intake |
|  | 103070 | Bacon intake |
|  | 103080 | Ham intake |
|  | 103090 | Liver intake |
|  | 102970 | Scotch egg intake |
| **Sweetened beverages** | 100170 | Fizzy drink intake |
|  | 100180 | Squash intake |
|  | 100190 | Orange juice intake |

# Supplementary Table 4 Missing number for brain imaging-derived phenotypes.

| **Phenotype** | **Field ID** | **Modality** | **Missing** |
| --- | --- | --- | --- |
| Volumetric scaling from T1 head image to standard space | 25000 | T1-weighted MRI | 3 |
| Volume of peripheral cortical grey matter (normalized for head size) | 25001 | T1-weighted MRI | 3 |
| Volume of peripheral cortical grey matter | 25002 | T1-weighted MRI | 3 |
| Volume of ventricular cerebrospinal fluid (normalized for head size) | 25003 | T1-weighted MRI | 3 |
| Volume of ventricular cerebrospinal fluid | 25004 | T1-weighted MRI | 3 |
| Volume of grey matter (normalized for head size) | 25005 | T1-weighted MRI | 3 |
| Volume of grey matter | 25006 | T1-weighted MRI | 3 |
| Volume of white matter (normalized for head size) | 25007 | T1-weighted MRI | 3 |
| Volume of white matter | 25008 | T1-weighted MRI | 3 |
| Volume of brain, grey+white matter (normalized for head size) | 25009 | T1-weighted MRI | 3 |
| Volume of brain, grey+white matter | 25010 | T1-weighted MRI | 3 |
| Volume of thalamus (left) | 25011 | T1-weighted MRI | 20 |
| Volume of thalamus (right) | 25012 | T1-weighted MRI | 20 |
| Volume of caudate (left) | 25013 | T1-weighted MRI | 20 |
| Volume of caudate (right) | 25014 | T1-weighted MRI | 20 |
| Volume of putamen (left) | 25015 | T1-weighted MRI | 20 |
| Volume of putamen (right) | 25016 | T1-weighted MRI | 20 |
| Volume of pallidum (left) | 25017 | T1-weighted MRI | 20 |
| Volume of pallidum (right) | 25018 | T1-weighted MRI | 20 |
| Volume of hippocampus (left) | 25019 | T1-weighted MRI | 20 |
| Volume of hippocampus (right) | 25020 | T1-weighted MRI | 20 |
| Volume of amygdala (left) | 25021 | T1-weighted MRI | 20 |
| Volume of amygdala (right) | 25022 | T1-weighted MRI | 20 |
| Volume of accumbens (left) | 25023 | T1-weighted MRI | 20 |
| Volume of accumbens (right) | 25024 | T1-weighted MRI | 20 |
| Volume of brain stem + 4th ventricle | 25025 | T1-weighted MRI | 20 |
| Volume of grey matter in Frontal Pole (left) | 25782 | T1-weighted MRI | 8 |
| Volume of grey matter in Frontal Pole (right) | 25783 | T1-weighted MRI | 8 |
| Volume of grey matter in Insular Cortex (left) | 25784 | T1-weighted MRI | 8 |
| Volume of grey matter in Insular Cortex (right) | 25785 | T1-weighted MRI | 8 |
| Volume of grey matter in Superior Frontal Gyrus (left) | 25786 | T1-weighted MRI | 8 |
| Volume of grey matter in Superior Frontal Gyrus (right) | 25787 | T1-weighted MRI | 8 |
| Volume of grey matter in Middle Frontal Gyrus (left) | 25788 | T1-weighted MRI | 8 |
| Volume of grey matter in Middle Frontal Gyrus (right) | 25789 | T1-weighted MRI | 8 |
| Volume of grey matter in Inferior Frontal Gyrus, pars triangularis (left) | 25790 | T1-weighted MRI | 8 |
| Volume of grey matter in Inferior Frontal Gyrus, pars triangularis (right) | 25791 | T1-weighted MRI | 8 |
| Volume of grey matter in Inferior Frontal Gyrus, pars opercularis (left) | 25792 | T1-weighted MRI | 8 |
| Volume of grey matter in Inferior Frontal Gyrus, pars opercularis (right) | 25793 | T1-weighted MRI | 8 |
| Volume of grey matter in Precentral Gyrus (left) | 25794 | T1-weighted MRI | 8 |
| Volume of grey matter in Precentral Gyrus (right) | 25795 | T1-weighted MRI | 8 |
| Volume of grey matter in Temporal Pole (left) | 25796 | T1-weighted MRI | 8 |
| Volume of grey matter in Temporal Pole (right) | 25797 | T1-weighted MRI | 8 |
| Volume of grey matter in Superior Temporal Gyrus, anterior division (left) | 25798 | T1-weighted MRI | 8 |
| Volume of grey matter in Superior Temporal Gyrus, anterior division (right) | 25799 | T1-weighted MRI | 8 |
| Volume of grey matter in Superior Temporal Gyrus, posterior division (left) | 25800 | T1-weighted MRI | 8 |
| Volume of grey matter in Superior Temporal Gyrus, posterior division (right) | 25801 | T1-weighted MRI | 8 |
| Volume of grey matter in Middle Temporal Gyrus, anterior division (left) | 25802 | T1-weighted MRI | 8 |
| Volume of grey matter in Middle Temporal Gyrus, anterior division (right) | 25803 | T1-weighted MRI | 8 |
| Volume of grey matter in Middle Temporal Gyrus, posterior division (left) | 25804 | T1-weighted MRI | 8 |
| Volume of grey matter in Middle Temporal Gyrus, posterior division (right) | 25805 | T1-weighted MRI | 8 |
| Volume of grey matter in Middle Temporal Gyrus, temporooccipital part (left) | 25806 | T1-weighted MRI | 8 |
| Volume of grey matter in Middle Temporal Gyrus, temporooccipital part (right) | 25807 | T1-weighted MRI | 8 |
| Volume of grey matter in Inferior Temporal Gyrus, anterior division (left) | 25808 | T1-weighted MRI | 8 |
| Volume of grey matter in Inferior Temporal Gyrus, anterior division (right) | 25809 | T1-weighted MRI | 8 |
| Volume of grey matter in Inferior Temporal Gyrus, posterior division (left) | 25810 | T1-weighted MRI | 8 |
| Volume of grey matter in Inferior Temporal Gyrus, posterior division (right) | 25811 | T1-weighted MRI | 8 |
| Volume of grey matter in Inferior Temporal Gyrus, temporooccipital part (left) | 25812 | T1-weighted MRI | 8 |
| Volume of grey matter in Inferior Temporal Gyrus, temporooccipital part (right) | 25813 | T1-weighted MRI | 8 |
| Volume of grey matter in Postcentral Gyrus (left) | 25814 | T1-weighted MRI | 8 |
| Volume of grey matter in Postcentral Gyrus (right) | 25815 | T1-weighted MRI | 8 |
| Volume of grey matter in Superior Parietal Lobule (left) | 25816 | T1-weighted MRI | 8 |
| Volume of grey matter in Superior Parietal Lobule (right) | 25817 | T1-weighted MRI | 8 |
| Volume of grey matter in Supramarginal Gyrus, anterior division (left) | 25818 | T1-weighted MRI | 8 |
| Volume of grey matter in Supramarginal Gyrus, anterior division (right) | 25819 | T1-weighted MRI | 8 |
| Volume of grey matter in Supramarginal Gyrus, posterior division (left) | 25820 | T1-weighted MRI | 8 |
| Volume of grey matter in Supramarginal Gyrus, posterior division (right) | 25821 | T1-weighted MRI | 8 |
| Volume of grey matter in Angular Gyrus (left) | 25822 | T1-weighted MRI | 8 |
| Volume of grey matter in Angular Gyrus (right) | 25823 | T1-weighted MRI | 8 |
| Volume of grey matter in Lateral Occipital Cortex, superior division (left) | 25824 | T1-weighted MRI | 8 |
| Volume of grey matter in Lateral Occipital Cortex, superior division (right) | 25825 | T1-weighted MRI | 8 |
| Volume of grey matter in Lateral Occipital Cortex, inferior division (left) | 25826 | T1-weighted MRI | 8 |
| Volume of grey matter in Lateral Occipital Cortex, inferior division (right) | 25827 | T1-weighted MRI | 8 |
| Volume of grey matter in Intracalcarine Cortex (left) | 25828 | T1-weighted MRI | 8 |
| Volume of grey matter in Intracalcarine Cortex (right) | 25829 | T1-weighted MRI | 8 |
| Volume of grey matter in Frontal Medial Cortex (left) | 25830 | T1-weighted MRI | 8 |
| Volume of grey matter in Frontal Medial Cortex (right) | 25831 | T1-weighted MRI | 8 |
| Volume of grey matter in Juxtapositional Lobule Cortex (formerly Supplementary Motor Cortex) (left) | 25832 | T1-weighted MRI | 8 |
| Volume of grey matter in Juxtapositional Lobule Cortex (formerly Supplementary Motor Cortex) (right) | 25833 | T1-weighted MRI | 8 |
| Volume of grey matter in Subcallosal Cortex (left) | 25834 | T1-weighted MRI | 8 |
| Volume of grey matter in Subcallosal Cortex (right) | 25835 | T1-weighted MRI | 8 |
| Volume of grey matter in Paracingulate Gyrus (left) | 25836 | T1-weighted MRI | 8 |
| Volume of grey matter in Paracingulate Gyrus (right) | 25837 | T1-weighted MRI | 8 |
| Volume of grey matter in Cingulate Gyrus, anterior division (left) | 25838 | T1-weighted MRI | 8 |
| Volume of grey matter in Cingulate Gyrus, anterior division (right) | 25839 | T1-weighted MRI | 8 |
| Volume of grey matter in Cingulate Gyrus, posterior division (left) | 25840 | T1-weighted MRI | 8 |
| Volume of grey matter in Cingulate Gyrus, posterior division (right) | 25841 | T1-weighted MRI | 8 |
| Volume of grey matter in Precuneous Cortex (left) | 25842 | T1-weighted MRI | 8 |
| Volume of grey matter in Precuneous Cortex (right) | 25843 | T1-weighted MRI | 8 |
| Volume of grey matter in Cuneal Cortex (left) | 25844 | T1-weighted MRI | 8 |
| Volume of grey matter in Cuneal Cortex (right) | 25845 | T1-weighted MRI | 8 |
| Volume of grey matter in Frontal Orbital Cortex (left) | 25846 | T1-weighted MRI | 8 |
| Volume of grey matter in Frontal Orbital Cortex (right) | 25847 | T1-weighted MRI | 8 |
| Volume of grey matter in Parahippocampal Gyrus, anterior division (left) | 25848 | T1-weighted MRI | 8 |
| Volume of grey matter in Parahippocampal Gyrus, anterior division (right) | 25849 | T1-weighted MRI | 8 |
| Volume of grey matter in Parahippocampal Gyrus, posterior division (left) | 25850 | T1-weighted MRI | 8 |
| Volume of grey matter in Parahippocampal Gyrus, posterior division (right) | 25851 | T1-weighted MRI | 8 |
| Volume of grey matter in Lingual Gyrus (left) | 25852 | T1-weighted MRI | 8 |
| Volume of grey matter in Lingual Gyrus (right) | 25853 | T1-weighted MRI | 8 |
| Volume of grey matter in Temporal Fusiform Cortex, anterior division (left) | 25854 | T1-weighted MRI | 8 |
| Volume of grey matter in Temporal Fusiform Cortex, anterior division (right) | 25855 | T1-weighted MRI | 8 |
| Volume of grey matter in Temporal Fusiform Cortex, posterior division (left) | 25856 | T1-weighted MRI | 8 |
| Volume of grey matter in Temporal Fusiform Cortex, posterior division (right) | 25857 | T1-weighted MRI | 8 |
| Volume of grey matter in Temporal Occipital Fusiform Cortex (left) | 25858 | T1-weighted MRI | 8 |
| Volume of grey matter in Temporal Occipital Fusiform Cortex (right) | 25859 | T1-weighted MRI | 8 |
| Volume of grey matter in Occipital Fusiform Gyrus (left) | 25860 | T1-weighted MRI | 8 |
| Volume of grey matter in Occipital Fusiform Gyrus (right) | 25861 | T1-weighted MRI | 8 |
| Volume of grey matter in Frontal Operculum Cortex (left) | 25862 | T1-weighted MRI | 8 |
| Volume of grey matter in Frontal Operculum Cortex (right) | 25863 | T1-weighted MRI | 8 |
| Volume of grey matter in Central Opercular Cortex (left) | 25864 | T1-weighted MRI | 8 |
| Volume of grey matter in Central Opercular Cortex (right) | 25865 | T1-weighted MRI | 8 |
| Volume of grey matter in Parietal Operculum Cortex (left) | 25866 | T1-weighted MRI | 8 |
| Volume of grey matter in Parietal Operculum Cortex (right) | 25867 | T1-weighted MRI | 8 |
| Volume of grey matter in Planum Polare (left) | 25868 | T1-weighted MRI | 8 |
| Volume of grey matter in Planum Polare (right) | 25869 | T1-weighted MRI | 8 |
| Volume of grey matter in Heschl's Gyrus (includes H1 and H2) (left) | 25870 | T1-weighted MRI | 8 |
| Volume of grey matter in Heschl's Gyrus (includes H1 and H2) (right) | 25871 | T1-weighted MRI | 8 |
| Volume of grey matter in Planum Temporale (left) | 25872 | T1-weighted MRI | 8 |
| Volume of grey matter in Planum Temporale (right) | 25873 | T1-weighted MRI | 8 |
| Volume of grey matter in Supracalcarine Cortex (left) | 25874 | T1-weighted MRI | 8 |
| Volume of grey matter in Supracalcarine Cortex (right) | 25875 | T1-weighted MRI | 8 |
| Volume of grey matter in Occipital Pole (left) | 25876 | T1-weighted MRI | 8 |
| Volume of grey matter in Occipital Pole (right) | 25877 | T1-weighted MRI | 8 |
| Volume of grey matter in Thalamus (left) | 25878 | T1-weighted MRI | 8 |
| Volume of grey matter in Thalamus (right) | 25879 | T1-weighted MRI | 8 |
| Volume of grey matter in Caudate (left) | 25880 | T1-weighted MRI | 8 |
| Volume of grey matter in Caudate (right) | 25881 | T1-weighted MRI | 8 |
| Volume of grey matter in Putamen (left) | 25882 | T1-weighted MRI | 8 |
| Volume of grey matter in Putamen (right) | 25883 | T1-weighted MRI | 8 |
| Volume of grey matter in Pallidum (left) | 25884 | T1-weighted MRI | 8 |
| Volume of grey matter in Pallidum (right) | 25885 | T1-weighted MRI | 8 |
| Volume of grey matter in Hippocampus (left) | 25886 | T1-weighted MRI | 8 |
| Volume of grey matter in Hippocampus (right) | 25887 | T1-weighted MRI | 8 |
| Volume of grey matter in Amygdala (left) | 25888 | T1-weighted MRI | 8 |
| Volume of grey matter in Amygdala (right) | 25889 | T1-weighted MRI | 8 |
| Volume of grey matter in Ventral Striatum (left) | 25890 | T1-weighted MRI | 8 |
| Volume of grey matter in Ventral Striatum (right) | 25891 | T1-weighted MRI | 8 |
| Volume of grey matter in Brain-Stem | 25892 | T1-weighted MRI | 8 |
| Volume of grey matter in I-IV Cerebellum (left) | 25893 | T1-weighted MRI | 8 |
| Volume of grey matter in I-IV Cerebellum (right) | 25894 | T1-weighted MRI | 8 |
| Volume of grey matter in V Cerebellum (left) | 25895 | T1-weighted MRI | 8 |
| Volume of grey matter in V Cerebellum (right) | 25896 | T1-weighted MRI | 8 |
| Volume of grey matter in VI Cerebellum (left) | 25897 | T1-weighted MRI | 8 |
| Volume of grey matter in VI Cerebellum (vermis) | 25898 | T1-weighted MRI | 8 |
| Volume of grey matter in VI Cerebellum (right) | 25899 | T1-weighted MRI | 8 |
| Volume of grey matter in Crus I Cerebellum (left) | 25900 | T1-weighted MRI | 8 |
| Volume of grey matter in Crus I Cerebellum (vermis) | 25901 | T1-weighted MRI | 8 |
| Volume of grey matter in Crus I Cerebellum (right) | 25902 | T1-weighted MRI | 8 |
| Volume of grey matter in Crus II Cerebellum (left) | 25903 | T1-weighted MRI | 8 |
| Volume of grey matter in Crus II Cerebellum (vermis) | 25904 | T1-weighted MRI | 8 |
| Volume of grey matter in Crus II Cerebellum (right) | 25905 | T1-weighted MRI | 8 |
| Volume of grey matter in VIIb Cerebellum (left) | 25906 | T1-weighted MRI | 8 |
| Volume of grey matter in VIIb Cerebellum (vermis) | 25907 | T1-weighted MRI | 8 |
| Volume of grey matter in VIIb Cerebellum (right) | 25908 | T1-weighted MRI | 8 |
| Volume of grey matter in VIIIa Cerebellum (left) | 25909 | T1-weighted MRI | 8 |
| Volume of grey matter in VIIIa Cerebellum (vermis) | 25910 | T1-weighted MRI | 8 |
| Volume of grey matter in VIIIa Cerebellum (right) | 25911 | T1-weighted MRI | 8 |
| Volume of grey matter in VIIIb Cerebellum (left) | 25912 | T1-weighted MRI | 8 |
| Volume of grey matter in VIIIb Cerebellum (vermis) | 25913 | T1-weighted MRI | 8 |
| Volume of grey matter in VIIIb Cerebellum (right) | 25914 | T1-weighted MRI | 8 |
| Volume of grey matter in IX Cerebellum (left) | 25915 | T1-weighted MRI | 8 |
| Volume of grey matter in IX Cerebellum (vermis) | 25916 | T1-weighted MRI | 8 |
| Volume of grey matter in IX Cerebellum (right) | 25917 | T1-weighted MRI | 8 |
| Volume of grey matter in X Cerebellum (left) | 25918 | T1-weighted MRI | 8 |
| Volume of grey matter in X Cerebellum (vermis) | 25919 | T1-weighted MRI | 8 |
| Volume of grey matter in X Cerebellum (right) | 25920 | T1-weighted MRI | 8 |
| Total volume of white matter hyperintensities (from T1 and T2_FLAIR images) | 25781 | T2-FLAIR | 1372 |
| Median T2star in thalamus (left) | 25026 | T2∗ | 4065 |
| Median T2star in thalamus (right) | 25027 | T2∗ | 4065 |
| Median T2star in caudate (left) | 25028 | T2∗ | 4065 |
| Median T2star in caudate (right) | 25029 | T2∗ | 4065 |
| Median T2star in putamen (left) | 25030 | T2∗ | 4065 |
| Median T2star in putamen (right) | 25031 | T2∗ | 4065 |
| Median T2star in pallidum (left) | 25032 | T2∗ | 4065 |
| Median T2star in pallidum (right) | 25033 | T2∗ | 4065 |
| Median T2star in hippocampus (left) | 25034 | T2∗ | 4065 |
| Median T2star in hippocampus (right) | 25035 | T2∗ | 4065 |
| Median T2star in amygdala (left) | 25036 | T2∗ | 4065 |
| Median T2star in amygdala (right) | 25037 | T2∗ | 4065 |
| Median T2star in accumbens (left) | 25038 | T2∗ | 4065 |
| Median T2star in accumbens (right) | 25039 | T2∗ | 4065 |
| Mean FA in middle cerebellar peduncle on FA skeleton | 25056 | diffusion-MRI | 2289 |
| Mean FA in pontine crossing tract on FA skeleton | 25057 | diffusion-MRI | 2289 |
| Mean FA in genu of corpus callosum on FA skeleton | 25058 | diffusion-MRI | 2289 |
| Mean FA in body of corpus callosum on FA skeleton | 25059 | diffusion-MRI | 2289 |
| Mean FA in splenium of corpus callosum on FA skeleton | 25060 | diffusion-MRI | 2289 |
| Mean FA in fornix on FA skeleton | 25061 | diffusion-MRI | 2289 |
| Mean FA in corticospinal tract on FA skeleton (right) | 25062 | diffusion-MRI | 2289 |
| Mean FA in corticospinal tract on FA skeleton (left) | 25063 | diffusion-MRI | 2289 |
| Mean FA in medial lemniscus on FA skeleton (right) | 25064 | diffusion-MRI | 2289 |
| Mean FA in medial lemniscus on FA skeleton (left) | 25065 | diffusion-MRI | 2289 |
| Mean FA in inferior cerebellar peduncle on FA skeleton (right) | 25066 | diffusion-MRI | 2289 |
| Mean FA in inferior cerebellar peduncle on FA skeleton (left) | 25067 | diffusion-MRI | 2289 |
| Mean FA in superior cerebellar peduncle on FA skeleton (right) | 25068 | diffusion-MRI | 2289 |
| Mean FA in superior cerebellar peduncle on FA skeleton (left) | 25069 | diffusion-MRI | 2289 |
| Mean FA in cerebral peduncle on FA skeleton (right) | 25070 | diffusion-MRI | 2289 |
| Mean FA in cerebral peduncle on FA skeleton (left) | 25071 | diffusion-MRI | 2289 |
| Mean FA in anterior limb of internal capsule on FA skeleton (right) | 25072 | diffusion-MRI | 2289 |
| Mean FA in anterior limb of internal capsule on FA skeleton (left) | 25073 | diffusion-MRI | 2289 |
| Mean FA in posterior limb of internal capsule on FA skeleton (right) | 25074 | diffusion-MRI | 2289 |
| Mean FA in posterior limb of internal capsule on FA skeleton (left) | 25075 | diffusion-MRI | 2289 |
| Mean FA in retrolenticular part of internal capsule on FA skeleton (right) | 25076 | diffusion-MRI | 2289 |
| Mean FA in retrolenticular part of internal capsule on FA skeleton (left) | 25077 | diffusion-MRI | 2289 |
| Mean FA in anterior corona radiata on FA skeleton (right) | 25078 | diffusion-MRI | 2289 |
| Mean FA in anterior corona radiata on FA skeleton (left) | 25079 | diffusion-MRI | 2289 |
| Mean FA in superior corona radiata on FA skeleton (right) | 25080 | diffusion-MRI | 2289 |
| Mean FA in superior corona radiata on FA skeleton (left) | 25081 | diffusion-MRI | 2289 |
| Mean FA in posterior corona radiata on FA skeleton (right) | 25082 | diffusion-MRI | 2289 |
| Mean FA in posterior corona radiata on FA skeleton (left) | 25083 | diffusion-MRI | 2289 |
| Mean FA in posterior thalamic radiation on FA skeleton (right) | 25084 | diffusion-MRI | 2289 |
| Mean FA in posterior thalamic radiation on FA skeleton (left) | 25085 | diffusion-MRI | 2289 |
| Mean FA in sagittal stratum on FA skeleton (right) | 25086 | diffusion-MRI | 2289 |
| Mean FA in sagittal stratum on FA skeleton (left) | 25087 | diffusion-MRI | 2289 |
| Mean FA in external capsule on FA skeleton (right) | 25088 | diffusion-MRI | 2289 |
| Mean FA in external capsule on FA skeleton (left) | 25089 | diffusion-MRI | 2289 |
| Mean FA in cingulum cingulate gyrus on FA skeleton (right) | 25090 | diffusion-MRI | 2289 |
| Mean FA in cingulum cingulate gyrus on FA skeleton (left) | 25091 | diffusion-MRI | 2289 |
| Mean FA in cingulum hippocampus on FA skeleton (right) | 25092 | diffusion-MRI | 2289 |
| Mean FA in cingulum hippocampus on FA skeleton (left) | 25093 | diffusion-MRI | 2289 |
| Mean FA in fornix cres+stria terminalis on FA skeleton (right) | 25094 | diffusion-MRI | 2289 |
| Mean FA in fornix cres+stria terminalis on FA skeleton (left) | 25095 | diffusion-MRI | 2289 |
| Mean FA in superior longitudinal fasciculus on FA skeleton (right) | 25096 | diffusion-MRI | 2289 |
| Mean FA in superior longitudinal fasciculus on FA skeleton (left) | 25097 | diffusion-MRI | 2289 |
| Mean FA in superior fronto-occipital fasciculus on FA skeleton (right) | 25098 | diffusion-MRI | 2289 |
| Mean FA in superior fronto-occipital fasciculus on FA skeleton (left) | 25099 | diffusion-MRI | 2289 |
| Mean FA in uncinate fasciculus on FA skeleton (right) | 25100 | diffusion-MRI | 2289 |
| Mean FA in uncinate fasciculus on FA skeleton (left) | 25101 | diffusion-MRI | 2289 |
| Mean FA in tapetum on FA skeleton (right) | 25102 | diffusion-MRI | 2289 |
| Mean FA in tapetum on FA skeleton (left) | 25103 | diffusion-MRI | 2289 |
| Mean MD in middle cerebellar peduncle on FA skeleton | 25104 | diffusion-MRI | 2289 |
| Mean MD in pontine crossing tract on FA skeleton | 25105 | diffusion-MRI | 2289 |
| Mean MD in genu of corpus callosum on FA skeleton | 25106 | diffusion-MRI | 2289 |
| Mean MD in body of corpus callosum on FA skeleton | 25107 | diffusion-MRI | 2289 |
| Mean MD in splenium of corpus callosum on FA skeleton | 25108 | diffusion-MRI | 2289 |
| Mean MD in fornix on FA skeleton | 25109 | diffusion-MRI | 2289 |
| Mean MD in corticospinal tract on FA skeleton (right) | 25110 | diffusion-MRI | 2289 |
| Mean MD in corticospinal tract on FA skeleton (left) | 25111 | diffusion-MRI | 2289 |
| Mean MD in medial lemniscus on FA skeleton (right) | 25112 | diffusion-MRI | 2289 |
| Mean MD in medial lemniscus on FA skeleton (left) | 25113 | diffusion-MRI | 2289 |
| Mean MD in inferior cerebellar peduncle on FA skeleton (right) | 25114 | diffusion-MRI | 2289 |
| Mean MD in inferior cerebellar peduncle on FA skeleton (left) | 25115 | diffusion-MRI | 2289 |
| Mean MD in superior cerebellar peduncle on FA skeleton (right) | 25116 | diffusion-MRI | 2289 |
| Mean MD in superior cerebellar peduncle on FA skeleton (left) | 25117 | diffusion-MRI | 2289 |
| Mean MD in cerebral peduncle on FA skeleton (right) | 25118 | diffusion-MRI | 2289 |
| Mean MD in cerebral peduncle on FA skeleton (left) | 25119 | diffusion-MRI | 2289 |
| Mean MD in anterior limb of internal capsule on FA skeleton (right) | 25120 | diffusion-MRI | 2289 |
| Mean MD in anterior limb of internal capsule on FA skeleton (left) | 25121 | diffusion-MRI | 2289 |
| Mean MD in posterior limb of internal capsule on FA skeleton (right) | 25122 | diffusion-MRI | 2289 |
| Mean MD in posterior limb of internal capsule on FA skeleton (left) | 25123 | diffusion-MRI | 2289 |
| Mean MD in retrolenticular part of internal capsule on FA skeleton (right) | 25124 | diffusion-MRI | 2289 |
| Mean MD in retrolenticular part of internal capsule on FA skeleton (left) | 25125 | diffusion-MRI | 2289 |
| Mean MD in anterior corona radiata on FA skeleton (right) | 25126 | diffusion-MRI | 2289 |
| Mean MD in anterior corona radiata on FA skeleton (left) | 25127 | diffusion-MRI | 2289 |
| Mean MD in superior corona radiata on FA skeleton (right) | 25128 | diffusion-MRI | 2289 |
| Mean MD in superior corona radiata on FA skeleton (left) | 25129 | diffusion-MRI | 2289 |
| Mean MD in posterior corona radiata on FA skeleton (right) | 25130 | diffusion-MRI | 2289 |
| Mean MD in posterior corona radiata on FA skeleton (left) | 25131 | diffusion-MRI | 2289 |
| Mean MD in posterior thalamic radiation on FA skeleton (right) | 25132 | diffusion-MRI | 2289 |
| Mean MD in posterior thalamic radiation on FA skeleton (left) | 25133 | diffusion-MRI | 2289 |
| Mean MD in sagittal stratum on FA skeleton (right) | 25134 | diffusion-MRI | 2289 |
| Mean MD in sagittal stratum on FA skeleton (left) | 25135 | diffusion-MRI | 2289 |
| Mean MD in external capsule on FA skeleton (right) | 25136 | diffusion-MRI | 2289 |
| Mean MD in external capsule on FA skeleton (left) | 25137 | diffusion-MRI | 2289 |
| Mean MD in cingulum cingulate gyrus on FA skeleton (right) | 25138 | diffusion-MRI | 2289 |
| Mean MD in cingulum cingulate gyrus on FA skeleton (left) | 25139 | diffusion-MRI | 2289 |
| Mean MD in cingulum hippocampus on FA skeleton (right) | 25140 | diffusion-MRI | 2289 |
| Mean MD in cingulum hippocampus on FA skeleton (left) | 25141 | diffusion-MRI | 2289 |
| Mean MD in fornix cres+stria terminalis on FA skeleton (right) | 25142 | diffusion-MRI | 2289 |
| Mean MD in fornix cres+stria terminalis on FA skeleton (left) | 25143 | diffusion-MRI | 2289 |
| Mean MD in superior longitudinal fasciculus on FA skeleton (right) | 25144 | diffusion-MRI | 2289 |
| Mean MD in superior longitudinal fasciculus on FA skeleton (left) | 25145 | diffusion-MRI | 2289 |
| Mean MD in superior fronto-occipital fasciculus on FA skeleton (right) | 25146 | diffusion-MRI | 2289 |
| Mean MD in superior fronto-occipital fasciculus on FA skeleton (left) | 25147 | diffusion-MRI | 2289 |
| Mean MD in uncinate fasciculus on FA skeleton (right) | 25148 | diffusion-MRI | 2289 |
| Mean MD in uncinate fasciculus on FA skeleton (left) | 25149 | diffusion-MRI | 2289 |
| Mean MD in tapetum on FA skeleton (right) | 25150 | diffusion-MRI | 2289 |
| Mean MD in tapetum on FA skeleton (left) | 25151 | diffusion-MRI | 2289 |
| Mean MO in middle cerebellar peduncle on FA skeleton | 25152 | diffusion-MRI | 2289 |
| Mean MO in pontine crossing tract on FA skeleton | 25153 | diffusion-MRI | 2289 |
| Mean MO in genu of corpus callosum on FA skeleton | 25154 | diffusion-MRI | 2289 |
| Mean MO in body of corpus callosum on FA skeleton | 25155 | diffusion-MRI | 2289 |
| Mean MO in splenium of corpus callosum on FA skeleton | 25156 | diffusion-MRI | 2289 |
| Mean MO in fornix on FA skeleton | 25157 | diffusion-MRI | 2289 |
| Mean MO in corticospinal tract on FA skeleton (right) | 25158 | diffusion-MRI | 2289 |
| Mean MO in corticospinal tract on FA skeleton (left) | 25159 | diffusion-MRI | 2289 |
| Mean MO in medial lemniscus on FA skeleton (right) | 25160 | diffusion-MRI | 2289 |
| Mean MO in medial lemniscus on FA skeleton (left) | 25161 | diffusion-MRI | 2289 |
| Mean MO in inferior cerebellar peduncle on FA skeleton (right) | 25162 | diffusion-MRI | 2289 |
| Mean MO in inferior cerebellar peduncle on FA skeleton (left) | 25163 | diffusion-MRI | 2289 |
| Mean MO in superior cerebellar peduncle on FA skeleton (right) | 25164 | diffusion-MRI | 2289 |
| Mean MO in superior cerebellar peduncle on FA skeleton (left) | 25165 | diffusion-MRI | 2289 |
| Mean MO in cerebral peduncle on FA skeleton (right) | 25166 | diffusion-MRI | 2289 |
| Mean MO in cerebral peduncle on FA skeleton (left) | 25167 | diffusion-MRI | 2289 |
| Mean MO in anterior limb of internal capsule on FA skeleton (right) | 25168 | diffusion-MRI | 2289 |
| Mean MO in anterior limb of internal capsule on FA skeleton (left) | 25169 | diffusion-MRI | 2289 |
| Mean MO in posterior limb of internal capsule on FA skeleton (right) | 25170 | diffusion-MRI | 2289 |
| Mean MO in posterior limb of internal capsule on FA skeleton (left) | 25171 | diffusion-MRI | 2289 |
| Mean MO in retrolenticular part of internal capsule on FA skeleton (right) | 25172 | diffusion-MRI | 2289 |
| Mean MO in retrolenticular part of internal capsule on FA skeleton (left) | 25173 | diffusion-MRI | 2289 |
| Mean MO in anterior corona radiata on FA skeleton (right) | 25174 | diffusion-MRI | 2289 |
| Mean MO in anterior corona radiata on FA skeleton (left) | 25175 | diffusion-MRI | 2289 |
| Mean MO in superior corona radiata on FA skeleton (right) | 25176 | diffusion-MRI | 2289 |
| Mean MO in superior corona radiata on FA skeleton (left) | 25177 | diffusion-MRI | 2289 |
| Mean MO in posterior corona radiata on FA skeleton (right) | 25178 | diffusion-MRI | 2289 |
| Mean MO in posterior corona radiata on FA skeleton (left) | 25179 | diffusion-MRI | 2289 |
| Mean MO in posterior thalamic radiation on FA skeleton (right) | 25180 | diffusion-MRI | 2289 |
| Mean MO in posterior thalamic radiation on FA skeleton (left) | 25181 | diffusion-MRI | 2289 |
| Mean MO in sagittal stratum on FA skeleton (right) | 25182 | diffusion-MRI | 2289 |
| Mean MO in sagittal stratum on FA skeleton (left) | 25183 | diffusion-MRI | 2289 |
| Mean MO in external capsule on FA skeleton (right) | 25184 | diffusion-MRI | 2289 |
| Mean MO in external capsule on FA skeleton (left) | 25185 | diffusion-MRI | 2289 |
| Mean MO in cingulum cingulate gyrus on FA skeleton (right) | 25186 | diffusion-MRI | 2289 |
| Mean MO in cingulum cingulate gyrus on FA skeleton (left) | 25187 | diffusion-MRI | 2289 |
| Mean MO in cingulum hippocampus on FA skeleton (right) | 25188 | diffusion-MRI | 2289 |
| Mean MO in cingulum hippocampus on FA skeleton (left) | 25189 | diffusion-MRI | 2289 |
| Mean MO in fornix cres+stria terminalis on FA skeleton (right) | 25190 | diffusion-MRI | 2289 |
| Mean MO in fornix cres+stria terminalis on FA skeleton (left) | 25191 | diffusion-MRI | 2289 |
| Mean MO in superior longitudinal fasciculus on FA skeleton (right) | 25192 | diffusion-MRI | 2289 |
| Mean MO in superior longitudinal fasciculus on FA skeleton (left) | 25193 | diffusion-MRI | 2289 |
| Mean MO in superior fronto-occipital fasciculus on FA skeleton (right) | 25194 | diffusion-MRI | 2289 |
| Mean MO in superior fronto-occipital fasciculus on FA skeleton (left) | 25195 | diffusion-MRI | 2289 |
| Mean MO in uncinate fasciculus on FA skeleton (right) | 25196 | diffusion-MRI | 2289 |
| Mean MO in uncinate fasciculus on FA skeleton (left) | 25197 | diffusion-MRI | 2289 |
| Mean MO in tapetum on FA skeleton (right) | 25198 | diffusion-MRI | 2289 |
| Mean MO in tapetum on FA skeleton (left) | 25199 | diffusion-MRI | 2289 |
| Mean L1 in middle cerebellar peduncle on FA skeleton | 25200 | diffusion-MRI | 2289 |
| Mean L1 in pontine crossing tract on FA skeleton | 25201 | diffusion-MRI | 2289 |
| Mean L1 in genu of corpus callosum on FA skeleton | 25202 | diffusion-MRI | 2289 |
| Mean L1 in body of corpus callosum on FA skeleton | 25203 | diffusion-MRI | 2289 |
| Mean L1 in splenium of corpus callosum on FA skeleton | 25204 | diffusion-MRI | 2289 |
| Mean L1 in fornix on FA skeleton | 25205 | diffusion-MRI | 2289 |
| Mean L1 in corticospinal tract on FA skeleton (right) | 25206 | diffusion-MRI | 2289 |
| Mean L1 in corticospinal tract on FA skeleton (left) | 25207 | diffusion-MRI | 2289 |
| Mean L1 in medial lemniscus on FA skeleton (right) | 25208 | diffusion-MRI | 2289 |
| Mean L1 in medial lemniscus on FA skeleton (left) | 25209 | diffusion-MRI | 2289 |
| Mean L1 in inferior cerebellar peduncle on FA skeleton (right) | 25210 | diffusion-MRI | 2289 |
| Mean L1 in inferior cerebellar peduncle on FA skeleton (left) | 25211 | diffusion-MRI | 2289 |
| Mean L1 in superior cerebellar peduncle on FA skeleton (right) | 25212 | diffusion-MRI | 2289 |
| Mean L1 in superior cerebellar peduncle on FA skeleton (left) | 25213 | diffusion-MRI | 2289 |
| Mean L1 in cerebral peduncle on FA skeleton (right) | 25214 | diffusion-MRI | 2289 |
| Mean L1 in cerebral peduncle on FA skeleton (left) | 25215 | diffusion-MRI | 2289 |
| Mean L1 in anterior limb of internal capsule on FA skeleton (right) | 25216 | diffusion-MRI | 2289 |
| Mean L1 in anterior limb of internal capsule on FA skeleton (left) | 25217 | diffusion-MRI | 2289 |
| Mean L1 in posterior limb of internal capsule on FA skeleton (right) | 25218 | diffusion-MRI | 2289 |
| Mean L1 in posterior limb of internal capsule on FA skeleton (left) | 25219 | diffusion-MRI | 2289 |
| Mean L1 in retrolenticular part of internal capsule on FA skeleton (right) | 25220 | diffusion-MRI | 2289 |
| Mean L1 in retrolenticular part of internal capsule on FA skeleton (left) | 25221 | diffusion-MRI | 2289 |
| Mean L1 in anterior corona radiata on FA skeleton (right) | 25222 | diffusion-MRI | 2289 |
| Mean L1 in anterior corona radiata on FA skeleton (left) | 25223 | diffusion-MRI | 2289 |
| Mean L1 in superior corona radiata on FA skeleton (right) | 25224 | diffusion-MRI | 2289 |
| Mean L1 in superior corona radiata on FA skeleton (left) | 25225 | diffusion-MRI | 2289 |
| Mean L1 in posterior corona radiata on FA skeleton (right) | 25226 | diffusion-MRI | 2289 |
| Mean L1 in posterior corona radiata on FA skeleton (left) | 25227 | diffusion-MRI | 2289 |
| Mean L1 in posterior thalamic radiation on FA skeleton (right) | 25228 | diffusion-MRI | 2289 |
| Mean L1 in posterior thalamic radiation on FA skeleton (left) | 25229 | diffusion-MRI | 2289 |
| Mean L1 in sagittal stratum on FA skeleton (right) | 25230 | diffusion-MRI | 2289 |
| Mean L1 in sagittal stratum on FA skeleton (left) | 25231 | diffusion-MRI | 2289 |
| Mean L1 in external capsule on FA skeleton (right) | 25232 | diffusion-MRI | 2289 |
| Mean L1 in external capsule on FA skeleton (left) | 25233 | diffusion-MRI | 2289 |
| Mean L1 in cingulum cingulate gyrus on FA skeleton (right) | 25234 | diffusion-MRI | 2289 |
| Mean L1 in cingulum cingulate gyrus on FA skeleton (left) | 25235 | diffusion-MRI | 2289 |
| Mean L1 in cingulum hippocampus on FA skeleton (right) | 25236 | diffusion-MRI | 2289 |
| Mean L1 in cingulum hippocampus on FA skeleton (left) | 25237 | diffusion-MRI | 2289 |
| Mean L1 in fornix cres+stria terminalis on FA skeleton (right) | 25238 | diffusion-MRI | 2289 |
| Mean L1 in fornix cres+stria terminalis on FA skeleton (left) | 25239 | diffusion-MRI | 2289 |
| Mean L1 in superior longitudinal fasciculus on FA skeleton (right) | 25240 | diffusion-MRI | 2289 |
| Mean L1 in superior longitudinal fasciculus on FA skeleton (left) | 25241 | diffusion-MRI | 2289 |
| Mean L1 in superior fronto-occipital fasciculus on FA skeleton (right) | 25242 | diffusion-MRI | 2289 |
| Mean L1 in superior fronto-occipital fasciculus on FA skeleton (left) | 25243 | diffusion-MRI | 2289 |
| Mean L1 in uncinate fasciculus on FA skeleton (right) | 25244 | diffusion-MRI | 2289 |
| Mean L1 in uncinate fasciculus on FA skeleton (left) | 25245 | diffusion-MRI | 2289 |
| Mean L1 in tapetum on FA skeleton (right) | 25246 | diffusion-MRI | 2289 |
| Mean L1 in tapetum on FA skeleton (left) | 25247 | diffusion-MRI | 2289 |
| Mean L2 in middle cerebellar peduncle on FA skeleton | 25248 | diffusion-MRI | 2289 |
| Mean L2 in pontine crossing tract on FA skeleton | 25249 | diffusion-MRI | 2289 |
| Mean L2 in genu of corpus callosum on FA skeleton | 25250 | diffusion-MRI | 2289 |
| Mean L2 in body of corpus callosum on FA skeleton | 25251 | diffusion-MRI | 2289 |
| Mean L2 in splenium of corpus callosum on FA skeleton | 25252 | diffusion-MRI | 2289 |
| Mean L2 in fornix on FA skeleton | 25253 | diffusion-MRI | 2289 |
| Mean L2 in corticospinal tract on FA skeleton (right) | 25254 | diffusion-MRI | 2289 |
| Mean L2 in corticospinal tract on FA skeleton (left) | 25255 | diffusion-MRI | 2289 |
| Mean L2 in medial lemniscus on FA skeleton (right) | 25256 | diffusion-MRI | 2289 |
| Mean L2 in medial lemniscus on FA skeleton (left) | 25257 | diffusion-MRI | 2289 |
| Mean L2 in inferior cerebellar peduncle on FA skeleton (right) | 25258 | diffusion-MRI | 2289 |
| Mean L2 in inferior cerebellar peduncle on FA skeleton (left) | 25259 | diffusion-MRI | 2289 |
| Mean L2 in superior cerebellar peduncle on FA skeleton (right) | 25260 | diffusion-MRI | 2289 |
| Mean L2 in superior cerebellar peduncle on FA skeleton (left) | 25261 | diffusion-MRI | 2289 |
| Mean L2 in cerebral peduncle on FA skeleton (right) | 25262 | diffusion-MRI | 2289 |
| Mean L2 in cerebral peduncle on FA skeleton (left) | 25263 | diffusion-MRI | 2289 |
| Mean L2 in anterior limb of internal capsule on FA skeleton (right) | 25264 | diffusion-MRI | 2289 |
| Mean L2 in anterior limb of internal capsule on FA skeleton (left) | 25265 | diffusion-MRI | 2289 |
| Mean L2 in posterior limb of internal capsule on FA skeleton (right) | 25266 | diffusion-MRI | 2289 |
| Mean L2 in posterior limb of internal capsule on FA skeleton (left) | 25267 | diffusion-MRI | 2289 |
| Mean L2 in retrolenticular part of internal capsule on FA skeleton (right) | 25268 | diffusion-MRI | 2289 |
| Mean L2 in retrolenticular part of internal capsule on FA skeleton (left) | 25269 | diffusion-MRI | 2289 |
| Mean L2 in anterior corona radiata on FA skeleton (right) | 25270 | diffusion-MRI | 2289 |
| Mean L2 in anterior corona radiata on FA skeleton (left) | 25271 | diffusion-MRI | 2289 |
| Mean L2 in superior corona radiata on FA skeleton (right) | 25272 | diffusion-MRI | 2289 |
| Mean L2 in superior corona radiata on FA skeleton (left) | 25273 | diffusion-MRI | 2289 |
| Mean L2 in posterior corona radiata on FA skeleton (right) | 25274 | diffusion-MRI | 2289 |
| Mean L2 in posterior corona radiata on FA skeleton (left) | 25275 | diffusion-MRI | 2289 |
| Mean L2 in posterior thalamic radiation on FA skeleton (right) | 25276 | diffusion-MRI | 2289 |
| Mean L2 in posterior thalamic radiation on FA skeleton (left) | 25277 | diffusion-MRI | 2289 |
| Mean L2 in sagittal stratum on FA skeleton (right) | 25278 | diffusion-MRI | 2289 |
| Mean L2 in sagittal stratum on FA skeleton (left) | 25279 | diffusion-MRI | 2289 |
| Mean L2 in external capsule on FA skeleton (right) | 25280 | diffusion-MRI | 2289 |
| Mean L2 in external capsule on FA skeleton (left) | 25281 | diffusion-MRI | 2289 |
| Mean L2 in cingulum cingulate gyrus on FA skeleton (right) | 25282 | diffusion-MRI | 2289 |
| Mean L2 in cingulum cingulate gyrus on FA skeleton (left) | 25283 | diffusion-MRI | 2289 |
| Mean L2 in cingulum hippocampus on FA skeleton (right) | 25284 | diffusion-MRI | 2289 |
| Mean L2 in cingulum hippocampus on FA skeleton (left) | 25285 | diffusion-MRI | 2289 |
| Mean L2 in fornix cres+stria terminalis on FA skeleton (right) | 25286 | diffusion-MRI | 2289 |
| Mean L2 in fornix cres+stria terminalis on FA skeleton (left) | 25287 | diffusion-MRI | 2289 |
| Mean L2 in superior longitudinal fasciculus on FA skeleton (right) | 25288 | diffusion-MRI | 2289 |
| Mean L2 in superior longitudinal fasciculus on FA skeleton (left) | 25289 | diffusion-MRI | 2289 |
| Mean L2 in superior fronto-occipital fasciculus on FA skeleton (right) | 25290 | diffusion-MRI | 2289 |
| Mean L2 in superior fronto-occipital fasciculus on FA skeleton (left) | 25291 | diffusion-MRI | 2289 |
| Mean L2 in uncinate fasciculus on FA skeleton (right) | 25292 | diffusion-MRI | 2289 |
| Mean L2 in uncinate fasciculus on FA skeleton (left) | 25293 | diffusion-MRI | 2289 |
| Mean L2 in tapetum on FA skeleton (right) | 25294 | diffusion-MRI | 2289 |
| Mean L2 in tapetum on FA skeleton (left) | 25295 | diffusion-MRI | 2289 |
| Mean L3 in middle cerebellar peduncle on FA skeleton | 25296 | diffusion-MRI | 2289 |
| Mean L3 in pontine crossing tract on FA skeleton | 25297 | diffusion-MRI | 2289 |
| Mean L3 in genu of corpus callosum on FA skeleton | 25298 | diffusion-MRI | 2289 |
| Mean L3 in body of corpus callosum on FA skeleton | 25299 | diffusion-MRI | 2289 |
| Mean L3 in splenium of corpus callosum on FA skeleton | 25300 | diffusion-MRI | 2289 |
| Mean L3 in fornix on FA skeleton | 25301 | diffusion-MRI | 2289 |
| Mean L3 in corticospinal tract on FA skeleton (right) | 25302 | diffusion-MRI | 2289 |
| Mean L3 in corticospinal tract on FA skeleton (left) | 25303 | diffusion-MRI | 2289 |
| Mean L3 in medial lemniscus on FA skeleton (right) | 25304 | diffusion-MRI | 2289 |
| Mean L3 in medial lemniscus on FA skeleton (left) | 25305 | diffusion-MRI | 2289 |
| Mean L3 in inferior cerebellar peduncle on FA skeleton (right) | 25306 | diffusion-MRI | 2289 |
| Mean L3 in inferior cerebellar peduncle on FA skeleton (left) | 25307 | diffusion-MRI | 2289 |
| Mean L3 in superior cerebellar peduncle on FA skeleton (right) | 25308 | diffusion-MRI | 2289 |
| Mean L3 in superior cerebellar peduncle on FA skeleton (left) | 25309 | diffusion-MRI | 2289 |
| Mean L3 in cerebral peduncle on FA skeleton (right) | 25310 | diffusion-MRI | 2289 |
| Mean L3 in cerebral peduncle on FA skeleton (left) | 25311 | diffusion-MRI | 2289 |
| Mean L3 in anterior limb of internal capsule on FA skeleton (right) | 25312 | diffusion-MRI | 2289 |
| Mean L3 in anterior limb of internal capsule on FA skeleton (left) | 25313 | diffusion-MRI | 2289 |
| Mean L3 in posterior limb of internal capsule on FA skeleton (right) | 25314 | diffusion-MRI | 2289 |
| Mean L3 in posterior limb of internal capsule on FA skeleton (left) | 25315 | diffusion-MRI | 2289 |
| Mean L3 in retrolenticular part of internal capsule on FA skeleton (right) | 25316 | diffusion-MRI | 2289 |
| Mean L3 in retrolenticular part of internal capsule on FA skeleton (left) | 25317 | diffusion-MRI | 2289 |
| Mean L3 in anterior corona radiata on FA skeleton (right) | 25318 | diffusion-MRI | 2289 |
| Mean L3 in anterior corona radiata on FA skeleton (left) | 25319 | diffusion-MRI | 2289 |
| Mean L3 in superior corona radiata on FA skeleton (right) | 25320 | diffusion-MRI | 2289 |
| Mean L3 in superior corona radiata on FA skeleton (left) | 25321 | diffusion-MRI | 2289 |
| Mean L3 in posterior corona radiata on FA skeleton (right) | 25322 | diffusion-MRI | 2289 |
| Mean L3 in posterior corona radiata on FA skeleton (left) | 25323 | diffusion-MRI | 2289 |
| Mean L3 in posterior thalamic radiation on FA skeleton (right) | 25324 | diffusion-MRI | 2289 |
| Mean L3 in posterior thalamic radiation on FA skeleton (left) | 25325 | diffusion-MRI | 2289 |
| Mean L3 in sagittal stratum on FA skeleton (right) | 25326 | diffusion-MRI | 2289 |
| Mean L3 in sagittal stratum on FA skeleton (left) | 25327 | diffusion-MRI | 2289 |
| Mean L3 in external capsule on FA skeleton (right) | 25328 | diffusion-MRI | 2289 |
| Mean L3 in external capsule on FA skeleton (left) | 25329 | diffusion-MRI | 2289 |
| Mean L3 in cingulum cingulate gyrus on FA skeleton (right) | 25330 | diffusion-MRI | 2289 |
| Mean L3 in cingulum cingulate gyrus on FA skeleton (left) | 25331 | diffusion-MRI | 2289 |
| Mean L3 in cingulum hippocampus on FA skeleton (right) | 25332 | diffusion-MRI | 2289 |
| Mean L3 in cingulum hippocampus on FA skeleton (left) | 25333 | diffusion-MRI | 2289 |
| Mean L3 in fornix cres+stria terminalis on FA skeleton (right) | 25334 | diffusion-MRI | 2289 |
| Mean L3 in fornix cres+stria terminalis on FA skeleton (left) | 25335 | diffusion-MRI | 2289 |
| Mean L3 in superior longitudinal fasciculus on FA skeleton (right) | 25336 | diffusion-MRI | 2289 |
| Mean L3 in superior longitudinal fasciculus on FA skeleton (left) | 25337 | diffusion-MRI | 2289 |
| Mean L3 in superior fronto-occipital fasciculus on FA skeleton (right) | 25338 | diffusion-MRI | 2289 |
| Mean L3 in superior fronto-occipital fasciculus on FA skeleton (left) | 25339 | diffusion-MRI | 2289 |
| Mean L3 in uncinate fasciculus on FA skeleton (right) | 25340 | diffusion-MRI | 2289 |
| Mean L3 in uncinate fasciculus on FA skeleton (left) | 25341 | diffusion-MRI | 2289 |
| Mean L3 in tapetum on FA skeleton (right) | 25342 | diffusion-MRI | 2289 |
| Mean L3 in tapetum on FA skeleton (left) | 25343 | diffusion-MRI | 2289 |
| Mean ICVF in middle cerebellar peduncle on FA skeleton | 25344 | diffusion-MRI | 2291 |
| Mean ICVF in pontine crossing tract on FA skeleton | 25345 | diffusion-MRI | 2291 |
| Mean ICVF in genu of corpus callosum on FA skeleton | 25346 | diffusion-MRI | 2291 |
| Mean ICVF in body of corpus callosum on FA skeleton | 25347 | diffusion-MRI | 2291 |
| Mean ICVF in splenium of corpus callosum on FA skeleton | 25348 | diffusion-MRI | 2291 |
| Mean ICVF in fornix on FA skeleton | 25349 | diffusion-MRI | 2291 |
| Mean ICVF in corticospinal tract on FA skeleton (right) | 25350 | diffusion-MRI | 2291 |
| Mean ICVF in corticospinal tract on FA skeleton (left) | 25351 | diffusion-MRI | 2291 |
| Mean ICVF in medial lemniscus on FA skeleton (right) | 25352 | diffusion-MRI | 2291 |
| Mean ICVF in medial lemniscus on FA skeleton (left) | 25353 | diffusion-MRI | 2291 |
| Mean ICVF in inferior cerebellar peduncle on FA skeleton (right) | 25354 | diffusion-MRI | 2291 |
| Mean ICVF in inferior cerebellar peduncle on FA skeleton (left) | 25355 | diffusion-MRI | 2291 |
| Mean ICVF in superior cerebellar peduncle on FA skeleton (right) | 25356 | diffusion-MRI | 2291 |
| Mean ICVF in superior cerebellar peduncle on FA skeleton (left) | 25357 | diffusion-MRI | 2291 |
| Mean ICVF in cerebral peduncle on FA skeleton (right) | 25358 | diffusion-MRI | 2291 |
| Mean ICVF in cerebral peduncle on FA skeleton (left) | 25359 | diffusion-MRI | 2291 |
| Mean ICVF in anterior limb of internal capsule on FA skeleton (right) | 25360 | diffusion-MRI | 2291 |
| Mean ICVF in anterior limb of internal capsule on FA skeleton (left) | 25361 | diffusion-MRI | 2291 |
| Mean ICVF in posterior limb of internal capsule on FA skeleton (right) | 25362 | diffusion-MRI | 2291 |
| Mean ICVF in posterior limb of internal capsule on FA skeleton (left) | 25363 | diffusion-MRI | 2291 |
| Mean ICVF in retrolenticular part of internal capsule on FA skeleton (right) | 25364 | diffusion-MRI | 2291 |
| Mean ICVF in retrolenticular part of internal capsule on FA skeleton (left) | 25365 | diffusion-MRI | 2291 |
| Mean ICVF in anterior corona radiata on FA skeleton (right) | 25366 | diffusion-MRI | 2291 |
| Mean ICVF in anterior corona radiata on FA skeleton (left) | 25367 | diffusion-MRI | 2291 |
| Mean ICVF in superior corona radiata on FA skeleton (right) | 25368 | diffusion-MRI | 2291 |
| Mean ICVF in superior corona radiata on FA skeleton (left) | 25369 | diffusion-MRI | 2291 |
| Mean ICVF in posterior corona radiata on FA skeleton (right) | 25370 | diffusion-MRI | 2291 |
| Mean ICVF in posterior corona radiata on FA skeleton (left) | 25371 | diffusion-MRI | 2291 |
| Mean ICVF in posterior thalamic radiation on FA skeleton (right) | 25372 | diffusion-MRI | 2291 |
| Mean ICVF in posterior thalamic radiation on FA skeleton (left) | 25373 | diffusion-MRI | 2291 |
| Mean ICVF in sagittal stratum on FA skeleton (right) | 25374 | diffusion-MRI | 2291 |
| Mean ICVF in sagittal stratum on FA skeleton (left) | 25375 | diffusion-MRI | 2291 |
| Mean ICVF in external capsule on FA skeleton (right) | 25376 | diffusion-MRI | 2291 |
| Mean ICVF in external capsule on FA skeleton (left) | 25377 | diffusion-MRI | 2291 |
| Mean ICVF in cingulum cingulate gyrus on FA skeleton (right) | 25378 | diffusion-MRI | 2291 |
| Mean ICVF in cingulum cingulate gyrus on FA skeleton (left) | 25379 | diffusion-MRI | 2291 |
| Mean ICVF in cingulum hippocampus on FA skeleton (right) | 25380 | diffusion-MRI | 2291 |
| Mean ICVF in cingulum hippocampus on FA skeleton (left) | 25381 | diffusion-MRI | 2291 |
| Mean ICVF in fornix cres+stria terminalis on FA skeleton (right) | 25382 | diffusion-MRI | 2291 |
| Mean ICVF in fornix cres+stria terminalis on FA skeleton (left) | 25383 | diffusion-MRI | 2291 |
| Mean ICVF in superior longitudinal fasciculus on FA skeleton (right) | 25384 | diffusion-MRI | 2291 |
| Mean ICVF in superior longitudinal fasciculus on FA skeleton (left) | 25385 | diffusion-MRI | 2291 |
| Mean ICVF in superior fronto-occipital fasciculus on FA skeleton (right) | 25386 | diffusion-MRI | 2291 |
| Mean ICVF in superior fronto-occipital fasciculus on FA skeleton (left) | 25387 | diffusion-MRI | 2291 |
| Mean ICVF in uncinate fasciculus on FA skeleton (right) | 25388 | diffusion-MRI | 2291 |
| Mean ICVF in uncinate fasciculus on FA skeleton (left) | 25389 | diffusion-MRI | 2291 |
| Mean ICVF in tapetum on FA skeleton (right) | 25390 | diffusion-MRI | 2291 |
| Mean ICVF in tapetum on FA skeleton (left) | 25391 | diffusion-MRI | 2291 |
| Mean OD in middle cerebellar peduncle on FA skeleton | 25392 | diffusion-MRI | 2291 |
| Mean OD in pontine crossing tract on FA skeleton | 25393 | diffusion-MRI | 2291 |
| Mean OD in genu of corpus callosum on FA skeleton | 25394 | diffusion-MRI | 2291 |
| Mean OD in body of corpus callosum on FA skeleton | 25395 | diffusion-MRI | 2291 |
| Mean OD in splenium of corpus callosum on FA skeleton | 25396 | diffusion-MRI | 2291 |
| Mean OD in fornix on FA skeleton | 25397 | diffusion-MRI | 2291 |
| Mean OD in corticospinal tract on FA skeleton (right) | 25398 | diffusion-MRI | 2291 |
| Mean OD in corticospinal tract on FA skeleton (left) | 25399 | diffusion-MRI | 2291 |
| Mean OD in medial lemniscus on FA skeleton (right) | 25400 | diffusion-MRI | 2291 |
| Mean OD in medial lemniscus on FA skeleton (left) | 25401 | diffusion-MRI | 2291 |
| Mean OD in inferior cerebellar peduncle on FA skeleton (right) | 25402 | diffusion-MRI | 2291 |
| Mean OD in inferior cerebellar peduncle on FA skeleton (left) | 25403 | diffusion-MRI | 2291 |
| Mean OD in superior cerebellar peduncle on FA skeleton (right) | 25404 | diffusion-MRI | 2291 |
| Mean OD in superior cerebellar peduncle on FA skeleton (left) | 25405 | diffusion-MRI | 2291 |
| Mean OD in cerebral peduncle on FA skeleton (right) | 25406 | diffusion-MRI | 2291 |
| Mean OD in cerebral peduncle on FA skeleton (left) | 25407 | diffusion-MRI | 2291 |
| Mean OD in anterior limb of internal capsule on FA skeleton (right) | 25408 | diffusion-MRI | 2291 |
| Mean OD in anterior limb of internal capsule on FA skeleton (left) | 25409 | diffusion-MRI | 2291 |
| Mean OD in posterior limb of internal capsule on FA skeleton (right) | 25410 | diffusion-MRI | 2291 |
| Mean OD in posterior limb of internal capsule on FA skeleton (left) | 25411 | diffusion-MRI | 2291 |
| Mean OD in retrolenticular part of internal capsule on FA skeleton (right) | 25412 | diffusion-MRI | 2291 |
| Mean OD in retrolenticular part of internal capsule on FA skeleton (left) | 25413 | diffusion-MRI | 2291 |
| Mean OD in anterior corona radiata on FA skeleton (right) | 25414 | diffusion-MRI | 2291 |
| Mean OD in anterior corona radiata on FA skeleton (left) | 25415 | diffusion-MRI | 2291 |
| Mean OD in superior corona radiata on FA skeleton (right) | 25416 | diffusion-MRI | 2291 |
| Mean OD in superior corona radiata on FA skeleton (left) | 25417 | diffusion-MRI | 2291 |
| Mean OD in posterior corona radiata on FA skeleton (right) | 25418 | diffusion-MRI | 2291 |
| Mean OD in posterior corona radiata on FA skeleton (left) | 25419 | diffusion-MRI | 2291 |
| Mean OD in posterior thalamic radiation on FA skeleton (right) | 25420 | diffusion-MRI | 2291 |
| Mean OD in posterior thalamic radiation on FA skeleton (left) | 25421 | diffusion-MRI | 2291 |
| Mean OD in sagittal stratum on FA skeleton (right) | 25422 | diffusion-MRI | 2291 |
| Mean OD in sagittal stratum on FA skeleton (left) | 25423 | diffusion-MRI | 2291 |
| Mean OD in external capsule on FA skeleton (right) | 25424 | diffusion-MRI | 2291 |
| Mean OD in external capsule on FA skeleton (left) | 25425 | diffusion-MRI | 2291 |
| Mean OD in cingulum cingulate gyrus on FA skeleton (right) | 25426 | diffusion-MRI | 2291 |
| Mean OD in cingulum cingulate gyrus on FA skeleton (left) | 25427 | diffusion-MRI | 2291 |
| Mean OD in cingulum hippocampus on FA skeleton (right) | 25428 | diffusion-MRI | 2291 |
| Mean OD in cingulum hippocampus on FA skeleton (left) | 25429 | diffusion-MRI | 2291 |
| Mean OD in fornix cres+stria terminalis on FA skeleton (right) | 25430 | diffusion-MRI | 2291 |
| Mean OD in fornix cres+stria terminalis on FA skeleton (left) | 25431 | diffusion-MRI | 2291 |
| Mean OD in superior longitudinal fasciculus on FA skeleton (right) | 25432 | diffusion-MRI | 2291 |
| Mean OD in superior longitudinal fasciculus on FA skeleton (left) | 25433 | diffusion-MRI | 2291 |
| Mean OD in superior fronto-occipital fasciculus on FA skeleton (right) | 25434 | diffusion-MRI | 2291 |
| Mean OD in superior fronto-occipital fasciculus on FA skeleton (left) | 25435 | diffusion-MRI | 2291 |
| Mean OD in uncinate fasciculus on FA skeleton (right) | 25436 | diffusion-MRI | 2291 |
| Mean OD in uncinate fasciculus on FA skeleton (left) | 25437 | diffusion-MRI | 2291 |
| Mean OD in tapetum on FA skeleton (right) | 25438 | diffusion-MRI | 2291 |
| Mean OD in tapetum on FA skeleton (left) | 25439 | diffusion-MRI | 2291 |
| Mean ISOVF in middle cerebellar peduncle on FA skeleton | 25440 | diffusion-MRI | 2291 |
| Mean ISOVF in pontine crossing tract on FA skeleton | 25441 | diffusion-MRI | 2291 |
| Mean ISOVF in genu of corpus callosum on FA skeleton | 25442 | diffusion-MRI | 2291 |
| Mean ISOVF in body of corpus callosum on FA skeleton | 25443 | diffusion-MRI | 2291 |
| Mean ISOVF in splenium of corpus callosum on FA skeleton | 25444 | diffusion-MRI | 2291 |
| Mean ISOVF in fornix on FA skeleton | 25445 | diffusion-MRI | 2291 |
| Mean ISOVF in corticospinal tract on FA skeleton (right) | 25446 | diffusion-MRI | 2291 |
| Mean ISOVF in corticospinal tract on FA skeleton (left) | 25447 | diffusion-MRI | 2291 |
| Mean ISOVF in medial lemniscus on FA skeleton (right) | 25448 | diffusion-MRI | 2291 |
| Mean ISOVF in medial lemniscus on FA skeleton (left) | 25449 | diffusion-MRI | 2291 |
| Mean ISOVF in inferior cerebellar peduncle on FA skeleton (right) | 25450 | diffusion-MRI | 2291 |
| Mean ISOVF in inferior cerebellar peduncle on FA skeleton (left) | 25451 | diffusion-MRI | 2291 |
| Mean ISOVF in superior cerebellar peduncle on FA skeleton (right) | 25452 | diffusion-MRI | 2291 |
| Mean ISOVF in superior cerebellar peduncle on FA skeleton (left) | 25453 | diffusion-MRI | 2291 |
| Mean ISOVF in cerebral peduncle on FA skeleton (right) | 25454 | diffusion-MRI | 2291 |
| Mean ISOVF in cerebral peduncle on FA skeleton (left) | 25455 | diffusion-MRI | 2291 |
| Mean ISOVF in anterior limb of internal capsule on FA skeleton (right) | 25456 | diffusion-MRI | 2291 |
| Mean ISOVF in anterior limb of internal capsule on FA skeleton (left) | 25457 | diffusion-MRI | 2291 |
| Mean ISOVF in posterior limb of internal capsule on FA skeleton (right) | 25458 | diffusion-MRI | 2291 |
| Mean ISOVF in posterior limb of internal capsule on FA skeleton (left) | 25459 | diffusion-MRI | 2291 |
| Mean ISOVF in retrolenticular part of internal capsule on FA skeleton (right) | 25460 | diffusion-MRI | 2291 |
| Mean ISOVF in retrolenticular part of internal capsule on FA skeleton (left) | 25461 | diffusion-MRI | 2291 |
| Mean ISOVF in anterior corona radiata on FA skeleton (right) | 25462 | diffusion-MRI | 2291 |
| Mean ISOVF in anterior corona radiata on FA skeleton (left) | 25463 | diffusion-MRI | 2291 |
| Mean ISOVF in superior corona radiata on FA skeleton (right) | 25464 | diffusion-MRI | 2291 |
| Mean ISOVF in superior corona radiata on FA skeleton (left) | 25465 | diffusion-MRI | 2291 |
| Mean ISOVF in posterior corona radiata on FA skeleton (right) | 25466 | diffusion-MRI | 2291 |
| Mean ISOVF in posterior corona radiata on FA skeleton (left) | 25467 | diffusion-MRI | 2291 |
| Mean ISOVF in posterior thalamic radiation on FA skeleton (right) | 25468 | diffusion-MRI | 2291 |
| Mean ISOVF in posterior thalamic radiation on FA skeleton (left) | 25469 | diffusion-MRI | 2291 |
| Mean ISOVF in sagittal stratum on FA skeleton (right) | 25470 | diffusion-MRI | 2291 |
| Mean ISOVF in sagittal stratum on FA skeleton (left) | 25471 | diffusion-MRI | 2291 |
| Mean ISOVF in external capsule on FA skeleton (right) | 25472 | diffusion-MRI | 2291 |
| Mean ISOVF in external capsule on FA skeleton (left) | 25473 | diffusion-MRI | 2291 |
| Mean ISOVF in cingulum cingulate gyrus on FA skeleton (right) | 25474 | diffusion-MRI | 2291 |
| Mean ISOVF in cingulum cingulate gyrus on FA skeleton (left) | 25475 | diffusion-MRI | 2291 |
| Mean ISOVF in cingulum hippocampus on FA skeleton (right) | 25476 | diffusion-MRI | 2291 |
| Mean ISOVF in cingulum hippocampus on FA skeleton (left) | 25477 | diffusion-MRI | 2291 |
| Mean ISOVF in fornix cres+stria terminalis on FA skeleton (right) | 25478 | diffusion-MRI | 2291 |
| Mean ISOVF in fornix cres+stria terminalis on FA skeleton (left) | 25479 | diffusion-MRI | 2291 |
| Mean ISOVF in superior longitudinal fasciculus on FA skeleton (right) | 25480 | diffusion-MRI | 2291 |
| Mean ISOVF in superior longitudinal fasciculus on FA skeleton (left) | 25481 | diffusion-MRI | 2291 |
| Mean ISOVF in superior fronto-occipital fasciculus on FA skeleton (right) | 25482 | diffusion-MRI | 2291 |
| Mean ISOVF in superior fronto-occipital fasciculus on FA skeleton (left) | 25483 | diffusion-MRI | 2291 |
| Mean ISOVF in uncinate fasciculus on FA skeleton (right) | 25484 | diffusion-MRI | 2291 |
| Mean ISOVF in uncinate fasciculus on FA skeleton (left) | 25485 | diffusion-MRI | 2291 |
| Mean ISOVF in tapetum on FA skeleton (right) | 25486 | diffusion-MRI | 2291 |
| Mean ISOVF in tapetum on FA skeleton (left) | 25487 | diffusion-MRI | 2291 |
| Weighted-mean FA in tract acoustic radiation (left) | 25488 | diffusion-MRI | 2291 |
| Weighted-mean FA in tract acoustic radiation (right) | 25489 | diffusion-MRI | 2291 |
| Weighted-mean FA in tract anterior thalamic radiation (left) | 25490 | diffusion-MRI | 2291 |
| Weighted-mean FA in tract anterior thalamic radiation (right) | 25491 | diffusion-MRI | 2291 |
| Weighted-mean FA in tract cingulate gyrus part of cingulum (left) | 25492 | diffusion-MRI | 2291 |
| Weighted-mean FA in tract cingulate gyrus part of cingulum (right) | 25493 | diffusion-MRI | 2291 |
| Weighted-mean FA in tract parahippocampal part of cingulum (left) | 25494 | diffusion-MRI | 2291 |
| Weighted-mean FA in tract parahippocampal part of cingulum (right) | 25495 | diffusion-MRI | 2291 |
| Weighted-mean FA in tract corticospinal tract (left) | 25496 | diffusion-MRI | 2291 |
| Weighted-mean FA in tract corticospinal tract (right) | 25497 | diffusion-MRI | 2291 |
| Weighted-mean FA in tract forceps major | 25498 | diffusion-MRI | 2291 |
| Weighted-mean FA in tract forceps minor | 25499 | diffusion-MRI | 2291 |
| Weighted-mean FA in tract inferior fronto-occipital fasciculus (left) | 25500 | diffusion-MRI | 2291 |
| Weighted-mean FA in tract inferior fronto-occipital fasciculus (right) | 25501 | diffusion-MRI | 2291 |
| Weighted-mean FA in tract inferior longitudinal fasciculus (left) | 25502 | diffusion-MRI | 2291 |
| Weighted-mean FA in tract inferior longitudinal fasciculus (right) | 25503 | diffusion-MRI | 2291 |
| Weighted-mean FA in tract middle cerebellar peduncle | 25504 | diffusion-MRI | 2291 |
| Weighted-mean FA in tract medial lemniscus (left) | 25505 | diffusion-MRI | 2291 |
| Weighted-mean FA in tract medial lemniscus (right) | 25506 | diffusion-MRI | 2291 |
| Weighted-mean FA in tract posterior thalamic radiation (left) | 25507 | diffusion-MRI | 2291 |
| Weighted-mean FA in tract posterior thalamic radiation (right) | 25508 | diffusion-MRI | 2291 |
| Weighted-mean FA in tract superior longitudinal fasciculus (left) | 25509 | diffusion-MRI | 2291 |
| Weighted-mean FA in tract superior longitudinal fasciculus (right) | 25510 | diffusion-MRI | 2291 |
| Weighted-mean FA in tract superior thalamic radiation (left) | 25511 | diffusion-MRI | 2291 |
| Weighted-mean FA in tract superior thalamic radiation (right) | 25512 | diffusion-MRI | 2291 |
| Weighted-mean FA in tract uncinate fasciculus (left) | 25513 | diffusion-MRI | 2291 |
| Weighted-mean FA in tract uncinate fasciculus (right) | 25514 | diffusion-MRI | 2291 |
| Weighted-mean MD in tract acoustic radiation (left) | 25515 | diffusion-MRI | 2291 |
| Weighted-mean MD in tract acoustic radiation (right) | 25516 | diffusion-MRI | 2291 |
| Weighted-mean MD in tract anterior thalamic radiation (left) | 25517 | diffusion-MRI | 2291 |
| Weighted-mean MD in tract anterior thalamic radiation (right) | 25518 | diffusion-MRI | 2291 |
| Weighted-mean MD in tract cingulate gyrus part of cingulum (left) | 25519 | diffusion-MRI | 2291 |
| Weighted-mean MD in tract cingulate gyrus part of cingulum (right) | 25520 | diffusion-MRI | 2291 |
| Weighted-mean MD in tract parahippocampal part of cingulum (left) | 25521 | diffusion-MRI | 2291 |
| Weighted-mean MD in tract parahippocampal part of cingulum (right) | 25522 | diffusion-MRI | 2291 |
| Weighted-mean MD in tract corticospinal tract (left) | 25523 | diffusion-MRI | 2291 |
| Weighted-mean MD in tract corticospinal tract (right) | 25524 | diffusion-MRI | 2291 |
| Weighted-mean MD in tract forceps major | 25525 | diffusion-MRI | 2291 |
| Weighted-mean MD in tract forceps minor | 25526 | diffusion-MRI | 2291 |
| Weighted-mean MD in tract inferior fronto-occipital fasciculus (left) | 25527 | diffusion-MRI | 2291 |
| Weighted-mean MD in tract inferior fronto-occipital fasciculus (right) | 25528 | diffusion-MRI | 2291 |
| Weighted-mean MD in tract inferior longitudinal fasciculus (left) | 25529 | diffusion-MRI | 2291 |
| Weighted-mean MD in tract inferior longitudinal fasciculus (right) | 25530 | diffusion-MRI | 2291 |
| Weighted-mean MD in tract middle cerebellar peduncle | 25531 | diffusion-MRI | 2291 |
| Weighted-mean MD in tract medial lemniscus (left) | 25532 | diffusion-MRI | 2291 |
| Weighted-mean MD in tract medial lemniscus (right) | 25533 | diffusion-MRI | 2291 |
| Weighted-mean MD in tract posterior thalamic radiation (left) | 25534 | diffusion-MRI | 2291 |
| Weighted-mean MD in tract posterior thalamic radiation (right) | 25535 | diffusion-MRI | 2291 |
| Weighted-mean MD in tract superior longitudinal fasciculus (left) | 25536 | diffusion-MRI | 2291 |
| Weighted-mean MD in tract superior longitudinal fasciculus (right) | 25537 | diffusion-MRI | 2291 |
| Weighted-mean MD in tract superior thalamic radiation (left) | 25538 | diffusion-MRI | 2291 |
| Weighted-mean MD in tract superior thalamic radiation (right) | 25539 | diffusion-MRI | 2291 |
| Weighted-mean MD in tract uncinate fasciculus (left) | 25540 | diffusion-MRI | 2291 |
| Weighted-mean MD in tract uncinate fasciculus (right) | 25541 | diffusion-MRI | 2291 |
| Weighted-mean MO in tract acoustic radiation (left) | 25542 | diffusion-MRI | 2291 |
| Weighted-mean MO in tract acoustic radiation (right) | 25543 | diffusion-MRI | 2291 |
| Weighted-mean MO in tract anterior thalamic radiation (left) | 25544 | diffusion-MRI | 2291 |
| Weighted-mean MO in tract anterior thalamic radiation (right) | 25545 | diffusion-MRI | 2291 |
| Weighted-mean MO in tract cingulate gyrus part of cingulum (left) | 25546 | diffusion-MRI | 2291 |
| Weighted-mean MO in tract cingulate gyrus part of cingulum (right) | 25547 | diffusion-MRI | 2291 |
| Weighted-mean MO in tract parahippocampal part of cingulum (left) | 25548 | diffusion-MRI | 2291 |
| Weighted-mean MO in tract parahippocampal part of cingulum (right) | 25549 | diffusion-MRI | 2291 |
| Weighted-mean MO in tract corticospinal tract (left) | 25550 | diffusion-MRI | 2291 |
| Weighted-mean MO in tract corticospinal tract (right) | 25551 | diffusion-MRI | 2291 |
| Weighted-mean MO in tract forceps major | 25552 | diffusion-MRI | 2291 |
| Weighted-mean MO in tract forceps minor | 25553 | diffusion-MRI | 2291 |
| Weighted-mean MO in tract inferior fronto-occipital fasciculus (left) | 25554 | diffusion-MRI | 2291 |
| Weighted-mean MO in tract inferior fronto-occipital fasciculus (right) | 25555 | diffusion-MRI | 2291 |
| Weighted-mean MO in tract inferior longitudinal fasciculus (left) | 25556 | diffusion-MRI | 2291 |
| Weighted-mean MO in tract inferior longitudinal fasciculus (right) | 25557 | diffusion-MRI | 2291 |
| Weighted-mean MO in tract middle cerebellar peduncle | 25558 | diffusion-MRI | 2291 |
| Weighted-mean MO in tract medial lemniscus (left) | 25559 | diffusion-MRI | 2291 |
| Weighted-mean MO in tract medial lemniscus (right) | 25560 | diffusion-MRI | 2291 |
| Weighted-mean MO in tract posterior thalamic radiation (left) | 25561 | diffusion-MRI | 2291 |
| Weighted-mean MO in tract posterior thalamic radiation (right) | 25562 | diffusion-MRI | 2291 |
| Weighted-mean MO in tract superior longitudinal fasciculus (left) | 25563 | diffusion-MRI | 2291 |
| Weighted-mean MO in tract superior longitudinal fasciculus (right) | 25564 | diffusion-MRI | 2291 |
| Weighted-mean MO in tract superior thalamic radiation (left) | 25565 | diffusion-MRI | 2291 |
| Weighted-mean MO in tract superior thalamic radiation (right) | 25566 | diffusion-MRI | 2291 |
| Weighted-mean MO in tract uncinate fasciculus (left) | 25567 | diffusion-MRI | 2291 |
| Weighted-mean MO in tract uncinate fasciculus (right) | 25568 | diffusion-MRI | 2291 |
| Weighted-mean L1 in tract acoustic radiation (left) | 25569 | diffusion-MRI | 2291 |
| Weighted-mean L1 in tract acoustic radiation (right) | 25570 | diffusion-MRI | 2291 |
| Weighted-mean L1 in tract anterior thalamic radiation (left) | 25571 | diffusion-MRI | 2291 |
| Weighted-mean L1 in tract anterior thalamic radiation (right) | 25572 | diffusion-MRI | 2291 |
| Weighted-mean L1 in tract cingulate gyrus part of cingulum (left) | 25573 | diffusion-MRI | 2291 |
| Weighted-mean L1 in tract cingulate gyrus part of cingulum (right) | 25574 | diffusion-MRI | 2291 |
| Weighted-mean L1 in tract parahippocampal part of cingulum (left) | 25575 | diffusion-MRI | 2291 |
| Weighted-mean L1 in tract parahippocampal part of cingulum (right) | 25576 | diffusion-MRI | 2291 |
| Weighted-mean L1 in tract corticospinal tract (left) | 25577 | diffusion-MRI | 2291 |
| Weighted-mean L1 in tract corticospinal tract (right) | 25578 | diffusion-MRI | 2291 |
| Weighted-mean L1 in tract forceps major | 25579 | diffusion-MRI | 2291 |
| Weighted-mean L1 in tract forceps minor | 25580 | diffusion-MRI | 2291 |
| Weighted-mean L1 in tract inferior fronto-occipital fasciculus (left) | 25581 | diffusion-MRI | 2291 |
| Weighted-mean L1 in tract inferior fronto-occipital fasciculus (right) | 25582 | diffusion-MRI | 2291 |
| Weighted-mean L1 in tract inferior longitudinal fasciculus (left) | 25583 | diffusion-MRI | 2291 |
| Weighted-mean L1 in tract inferior longitudinal fasciculus (right) | 25584 | diffusion-MRI | 2291 |
| Weighted-mean L1 in tract middle cerebellar peduncle | 25585 | diffusion-MRI | 2291 |
| Weighted-mean L1 in tract medial lemniscus (left) | 25586 | diffusion-MRI | 2291 |
| Weighted-mean L1 in tract medial lemniscus (right) | 25587 | diffusion-MRI | 2291 |
| Weighted-mean L1 in tract posterior thalamic radiation (left) | 25588 | diffusion-MRI | 2291 |
| Weighted-mean L1 in tract posterior thalamic radiation (right) | 25589 | diffusion-MRI | 2291 |
| Weighted-mean L1 in tract superior longitudinal fasciculus (left) | 25590 | diffusion-MRI | 2291 |
| Weighted-mean L1 in tract superior longitudinal fasciculus (right) | 25591 | diffusion-MRI | 2291 |
| Weighted-mean L1 in tract superior thalamic radiation (left) | 25592 | diffusion-MRI | 2291 |
| Weighted-mean L1 in tract superior thalamic radiation (right) | 25593 | diffusion-MRI | 2291 |
| Weighted-mean L1 in tract uncinate fasciculus (left) | 25594 | diffusion-MRI | 2291 |
| Weighted-mean L1 in tract uncinate fasciculus (right) | 25595 | diffusion-MRI | 2291 |
| Weighted-mean L2 in tract acoustic radiation (left) | 25596 | diffusion-MRI | 2291 |
| Weighted-mean L2 in tract acoustic radiation (right) | 25597 | diffusion-MRI | 2291 |
| Weighted-mean L2 in tract anterior thalamic radiation (left) | 25598 | diffusion-MRI | 2291 |
| Weighted-mean L2 in tract anterior thalamic radiation (right) | 25599 | diffusion-MRI | 2291 |
| Weighted-mean L2 in tract cingulate gyrus part of cingulum (left) | 25600 | diffusion-MRI | 2291 |
| Weighted-mean L2 in tract cingulate gyrus part of cingulum (right) | 25601 | diffusion-MRI | 2291 |
| Weighted-mean L2 in tract parahippocampal part of cingulum (left) | 25602 | diffusion-MRI | 2291 |
| Weighted-mean L2 in tract parahippocampal part of cingulum (right) | 25603 | diffusion-MRI | 2291 |
| Weighted-mean L2 in tract corticospinal tract (left) | 25604 | diffusion-MRI | 2291 |
| Weighted-mean L2 in tract corticospinal tract (right) | 25605 | diffusion-MRI | 2291 |
| Weighted-mean L2 in tract forceps major | 25606 | diffusion-MRI | 2291 |
| Weighted-mean L2 in tract forceps minor | 25607 | diffusion-MRI | 2291 |
| Weighted-mean L2 in tract inferior fronto-occipital fasciculus (left) | 25608 | diffusion-MRI | 2291 |
| Weighted-mean L2 in tract inferior fronto-occipital fasciculus (right) | 25609 | diffusion-MRI | 2291 |
| Weighted-mean L2 in tract inferior longitudinal fasciculus (left) | 25610 | diffusion-MRI | 2291 |
| Weighted-mean L2 in tract inferior longitudinal fasciculus (right) | 25611 | diffusion-MRI | 2291 |
| Weighted-mean L2 in tract middle cerebellar peduncle | 25612 | diffusion-MRI | 2291 |
| Weighted-mean L2 in tract medial lemniscus (left) | 25613 | diffusion-MRI | 2291 |
| Weighted-mean L2 in tract medial lemniscus (right) | 25614 | diffusion-MRI | 2291 |
| Weighted-mean L2 in tract posterior thalamic radiation (left) | 25615 | diffusion-MRI | 2291 |
| Weighted-mean L2 in tract posterior thalamic radiation (right) | 25616 | diffusion-MRI | 2291 |
| Weighted-mean L2 in tract superior longitudinal fasciculus (left) | 25617 | diffusion-MRI | 2291 |
| Weighted-mean L2 in tract superior longitudinal fasciculus (right) | 25618 | diffusion-MRI | 2291 |
| Weighted-mean L2 in tract superior thalamic radiation (left) | 25619 | diffusion-MRI | 2291 |
| Weighted-mean L2 in tract superior thalamic radiation (right) | 25620 | diffusion-MRI | 2291 |
| Weighted-mean L2 in tract uncinate fasciculus (left) | 25621 | diffusion-MRI | 2291 |
| Weighted-mean L2 in tract uncinate fasciculus (right) | 25622 | diffusion-MRI | 2291 |
| Weighted-mean L3 in tract acoustic radiation (left) | 25623 | diffusion-MRI | 2291 |
| Weighted-mean L3 in tract acoustic radiation (right) | 25624 | diffusion-MRI | 2291 |
| Weighted-mean L3 in tract anterior thalamic radiation (left) | 25625 | diffusion-MRI | 2291 |
| Weighted-mean L3 in tract anterior thalamic radiation (right) | 25626 | diffusion-MRI | 2291 |
| Weighted-mean L3 in tract cingulate gyrus part of cingulum (left) | 25627 | diffusion-MRI | 2291 |
| Weighted-mean L3 in tract cingulate gyrus part of cingulum (right) | 25628 | diffusion-MRI | 2291 |
| Weighted-mean L3 in tract parahippocampal part of cingulum (left) | 25629 | diffusion-MRI | 2291 |
| Weighted-mean L3 in tract parahippocampal part of cingulum (right) | 25630 | diffusion-MRI | 2291 |
| Weighted-mean L3 in tract corticospinal tract (left) | 25631 | diffusion-MRI | 2291 |
| Weighted-mean L3 in tract corticospinal tract (right) | 25632 | diffusion-MRI | 2291 |
| Weighted-mean L3 in tract forceps major | 25633 | diffusion-MRI | 2291 |
| Weighted-mean L3 in tract forceps minor | 25634 | diffusion-MRI | 2291 |
| Weighted-mean L3 in tract inferior fronto-occipital fasciculus (left) | 25635 | diffusion-MRI | 2291 |
| Weighted-mean L3 in tract inferior fronto-occipital fasciculus (right) | 25636 | diffusion-MRI | 2291 |
| Weighted-mean L3 in tract inferior longitudinal fasciculus (left) | 25637 | diffusion-MRI | 2291 |
| Weighted-mean L3 in tract inferior longitudinal fasciculus (right) | 25638 | diffusion-MRI | 2291 |
| Weighted-mean L3 in tract middle cerebellar peduncle | 25639 | diffusion-MRI | 2291 |
| Weighted-mean L3 in tract medial lemniscus (left) | 25640 | diffusion-MRI | 2291 |
| Weighted-mean L3 in tract medial lemniscus (right) | 25641 | diffusion-MRI | 2291 |
| Weighted-mean L3 in tract posterior thalamic radiation (left) | 25642 | diffusion-MRI | 2291 |
| Weighted-mean L3 in tract posterior thalamic radiation (right) | 25643 | diffusion-MRI | 2291 |
| Weighted-mean L3 in tract superior longitudinal fasciculus (left) | 25644 | diffusion-MRI | 2291 |
| Weighted-mean L3 in tract superior longitudinal fasciculus (right) | 25645 | diffusion-MRI | 2291 |
| Weighted-mean L3 in tract superior thalamic radiation (left) | 25646 | diffusion-MRI | 2291 |
| Weighted-mean L3 in tract superior thalamic radiation (right) | 25647 | diffusion-MRI | 2291 |
| Weighted-mean L3 in tract uncinate fasciculus (left) | 25648 | diffusion-MRI | 2291 |
| Weighted-mean L3 in tract uncinate fasciculus (right) | 25649 | diffusion-MRI | 2291 |
| Weighted-mean ICVF in tract acoustic radiation (left) | 25650 | diffusion-MRI | 2292 |
| Weighted-mean ICVF in tract acoustic radiation (right) | 25651 | diffusion-MRI | 2292 |
| Weighted-mean ICVF in tract anterior thalamic radiation (left) | 25652 | diffusion-MRI | 2292 |
| Weighted-mean ICVF in tract anterior thalamic radiation (right) | 25653 | diffusion-MRI | 2292 |
| Weighted-mean ICVF in tract cingulate gyrus part of cingulum (left) | 25654 | diffusion-MRI | 2292 |
| Weighted-mean ICVF in tract cingulate gyrus part of cingulum (right) | 25655 | diffusion-MRI | 2292 |
| Weighted-mean ICVF in tract parahippocampal part of cingulum (left) | 25656 | diffusion-MRI | 2292 |
| Weighted-mean ICVF in tract parahippocampal part of cingulum (right) | 25657 | diffusion-MRI | 2292 |
| Weighted-mean ICVF in tract corticospinal tract (left) | 25658 | diffusion-MRI | 2292 |
| Weighted-mean ICVF in tract corticospinal tract (right) | 25659 | diffusion-MRI | 2292 |
| Weighted-mean ICVF in tract forceps major | 25660 | diffusion-MRI | 2292 |
| Weighted-mean ICVF in tract forceps minor | 25661 | diffusion-MRI | 2292 |
| Weighted-mean ICVF in tract inferior fronto-occipital fasciculus (left) | 25662 | diffusion-MRI | 2292 |
| Weighted-mean ICVF in tract inferior fronto-occipital fasciculus (right) | 25663 | diffusion-MRI | 2292 |
| Weighted-mean ICVF in tract inferior longitudinal fasciculus (left) | 25664 | diffusion-MRI | 2292 |
| Weighted-mean ICVF in tract inferior longitudinal fasciculus (right) | 25665 | diffusion-MRI | 2292 |
| Weighted-mean ICVF in tract middle cerebellar peduncle | 25666 | diffusion-MRI | 2292 |
| Weighted-mean ICVF in tract medial lemniscus (left) | 25667 | diffusion-MRI | 2292 |
| Weighted-mean ICVF in tract medial lemniscus (right) | 25668 | diffusion-MRI | 2292 |
| Weighted-mean ICVF in tract posterior thalamic radiation (left) | 25669 | diffusion-MRI | 2292 |
| Weighted-mean ICVF in tract posterior thalamic radiation (right) | 25670 | diffusion-MRI | 2292 |
| Weighted-mean ICVF in tract superior longitudinal fasciculus (left) | 25671 | diffusion-MRI | 2292 |
| Weighted-mean ICVF in tract superior longitudinal fasciculus (right) | 25672 | diffusion-MRI | 2292 |
| Weighted-mean ICVF in tract superior thalamic radiation (left) | 25673 | diffusion-MRI | 2292 |
| Weighted-mean ICVF in tract superior thalamic radiation (right) | 25674 | diffusion-MRI | 2292 |
| Weighted-mean ICVF in tract uncinate fasciculus (left) | 25675 | diffusion-MRI | 2292 |
| Weighted-mean ICVF in tract uncinate fasciculus (right) | 25676 | diffusion-MRI | 2292 |
| Weighted-mean OD in tract acoustic radiation (left) | 25677 | diffusion-MRI | 2292 |
| Weighted-mean OD in tract acoustic radiation (right) | 25678 | diffusion-MRI | 2292 |
| Weighted-mean OD in tract anterior thalamic radiation (left) | 25679 | diffusion-MRI | 2292 |
| Weighted-mean OD in tract anterior thalamic radiation (right) | 25680 | diffusion-MRI | 2292 |
| Weighted-mean OD in tract cingulate gyrus part of cingulum (left) | 25681 | diffusion-MRI | 2292 |
| Weighted-mean OD in tract cingulate gyrus part of cingulum (right) | 25682 | diffusion-MRI | 2292 |
| Weighted-mean OD in tract parahippocampal part of cingulum (left) | 25683 | diffusion-MRI | 2292 |
| Weighted-mean OD in tract parahippocampal part of cingulum (right) | 25684 | diffusion-MRI | 2292 |
| Weighted-mean OD in tract corticospinal tract (left) | 25685 | diffusion-MRI | 2292 |
| Weighted-mean OD in tract corticospinal tract (right) | 25686 | diffusion-MRI | 2292 |
| Weighted-mean OD in tract forceps major | 25687 | diffusion-MRI | 2292 |
| Weighted-mean OD in tract forceps minor | 25688 | diffusion-MRI | 2292 |
| Weighted-mean OD in tract inferior fronto-occipital fasciculus (left) | 25689 | diffusion-MRI | 2292 |
| Weighted-mean OD in tract inferior fronto-occipital fasciculus (right) | 25690 | diffusion-MRI | 2292 |
| Weighted-mean OD in tract inferior longitudinal fasciculus (left) | 25691 | diffusion-MRI | 2292 |
| Weighted-mean OD in tract inferior longitudinal fasciculus (right) | 25692 | diffusion-MRI | 2292 |
| Weighted-mean OD in tract middle cerebellar peduncle | 25693 | diffusion-MRI | 2292 |
| Weighted-mean OD in tract medial lemniscus (left) | 25694 | diffusion-MRI | 2292 |
| Weighted-mean OD in tract medial lemniscus (right) | 25695 | diffusion-MRI | 2292 |
| Weighted-mean OD in tract posterior thalamic radiation (left) | 25696 | diffusion-MRI | 2292 |
| Weighted-mean OD in tract posterior thalamic radiation (right) | 25697 | diffusion-MRI | 2292 |
| Weighted-mean OD in tract superior longitudinal fasciculus (left) | 25698 | diffusion-MRI | 2292 |
| Weighted-mean OD in tract superior longitudinal fasciculus (right) | 25699 | diffusion-MRI | 2292 |
| Weighted-mean OD in tract superior thalamic radiation (left) | 25700 | diffusion-MRI | 2292 |
| Weighted-mean OD in tract superior thalamic radiation (right) | 25701 | diffusion-MRI | 2292 |
| Weighted-mean OD in tract uncinate fasciculus (left) | 25702 | diffusion-MRI | 2292 |
| Weighted-mean OD in tract uncinate fasciculus (right) | 25703 | diffusion-MRI | 2292 |
| Weighted-mean ISOVF in tract acoustic radiation (left) | 25704 | diffusion-MRI | 2292 |
| Weighted-mean ISOVF in tract acoustic radiation (right) | 25705 | diffusion-MRI | 2292 |
| Weighted-mean ISOVF in tract anterior thalamic radiation (left) | 25706 | diffusion-MRI | 2292 |
| Weighted-mean ISOVF in tract anterior thalamic radiation (right) | 25707 | diffusion-MRI | 2292 |
| Weighted-mean ISOVF in tract cingulate gyrus part of cingulum (left) | 25708 | diffusion-MRI | 2292 |
| Weighted-mean ISOVF in tract cingulate gyrus part of cingulum (right) | 25709 | diffusion-MRI | 2292 |
| Weighted-mean ISOVF in tract parahippocampal part of cingulum (left) | 25710 | diffusion-MRI | 2292 |
| Weighted-mean ISOVF in tract parahippocampal part of cingulum (right) | 25711 | diffusion-MRI | 2292 |
| Weighted-mean ISOVF in tract corticospinal tract (left) | 25712 | diffusion-MRI | 2292 |
| Weighted-mean ISOVF in tract corticospinal tract (right) | 25713 | diffusion-MRI | 2292 |
| Weighted-mean ISOVF in tract forceps major | 25714 | diffusion-MRI | 2292 |
| Weighted-mean ISOVF in tract forceps minor | 25715 | diffusion-MRI | 2292 |
| Weighted-mean ISOVF in tract inferior fronto-occipital fasciculus (left) | 25716 | diffusion-MRI | 2292 |
| Weighted-mean ISOVF in tract inferior fronto-occipital fasciculus (right) | 25717 | diffusion-MRI | 2292 |
| Weighted-mean ISOVF in tract inferior longitudinal fasciculus (left) | 25718 | diffusion-MRI | 2292 |
| Weighted-mean ISOVF in tract inferior longitudinal fasciculus (right) | 25719 | diffusion-MRI | 2292 |
| Weighted-mean ISOVF in tract middle cerebellar peduncle | 25720 | diffusion-MRI | 2292 |
| Weighted-mean ISOVF in tract medial lemniscus (left) | 25721 | diffusion-MRI | 2292 |
| Weighted-mean ISOVF in tract medial lemniscus (right) | 25722 | diffusion-MRI | 2292 |
| Weighted-mean ISOVF in tract posterior thalamic radiation (left) | 25723 | diffusion-MRI | 2292 |
| Weighted-mean ISOVF in tract posterior thalamic radiation (right) | 25724 | diffusion-MRI | 2292 |
| Weighted-mean ISOVF in tract superior longitudinal fasciculus (left) | 25725 | diffusion-MRI | 2292 |
| Weighted-mean ISOVF in tract superior longitudinal fasciculus (right) | 25726 | diffusion-MRI | 2292 |
| Weighted-mean ISOVF in tract superior thalamic radiation (left) | 25727 | diffusion-MRI | 2292 |
| Weighted-mean ISOVF in tract superior thalamic radiation (right) | 25728 | diffusion-MRI | 2292 |
| Weighted-mean ISOVF in tract uncinate fasciculus (left) | 25729 | diffusion-MRI | 2292 |
| Weighted-mean ISOVF in tract uncinate fasciculus (right) | 25730 | diffusion-MRI | 2292 |
| rfMRI partial correlation matrix, dimension 25 | 25752^*^ | resting-state fMRI | 1888 |
| Median BOLD effect (in group-defined mask) for shapes activation | 25040 | task fMRI | 7373 |
| Median z-statistic (in group-defined mask) for shapes activation | 25042 | task fMRI | 7373 |
| Median BOLD effect (in group-defined mask) for faces activation | 25044 | task fMRI | 7373 |
| Median z-statistic (in group-defined mask) for faces activation | 25046 | task fMRI | 7373 |
| Median BOLD effect (in group-defined mask) for faces-shapes contrast | 25048 | task fMRI | 7373 |
| Median BOLD effect (in group-defined amygdala activation mask) for faces-shapes contrast | 25052 | task fMRI | 7373 |
| Median z-statistic (in group-defined amygdala activation mask) for faces-shapes contrast | 25054 | task fMRI | 7373 |
| 90th percentile of BOLD effect (in group-defined mask) for shapes activation | 25761 | task fMRI | 7373 |
| 90th percentile of z-statistic (in group-defined mask) for shapes activation | 25762 | task fMRI | 7373 |
| 90th percentile of BOLD effect (in group-defined mask) for faces activation | 25763 | task fMRI | 7373 |
| 90th percentile of z-statistic (in group-defined mask) for faces activation | 25764 | task fMRI | 7373 |
| 90th percentile of BOLD effect (in group-defined mask) for faces-shapes contrast | 25765 | task fMRI | 7373 |
| 90th percentile of BOLD effect (in group-defined amygdala activation mask) for faces-shapes contrast | 25767 | task fMRI | 7373 |
| 90th percentile of z-statistic (in group-defined amygdala activation mask) for faces-shapes contrast | 25768 | task fMRI | 7373 |

# Supplementary Table 5. The coefficient of each phenotype in Least Absolute Shrinkage and Selection Operator (LASSO) regression without feature selection.

| **Phenotype^1^** | **Coefficient** |
| --- | --- |
| Volume of grey matter (normalized for head size) | -1.3516 |
| Weighted-mean ICVF in tract forceps minor | -0.7169 |
| Mean ISOVF in fornix on FA skeleton | 0.6877 |
| Volume of grey matter in Ventral Striatum (left) | -0.6099 |
| Mean FA in superior cerebellar peduncle on FA skeleton (right) | 0.5779 |
| Volume of brain stem + 4th ventricle | 0.5745 |
| Weighted-mean OD in tract anterior thalamic radiation (right) | -0.5529 |
| Mean L1 in anterior limb of internal capsule on FA skeleton (right) | 0.5410 |
| Volume of thalamus (right) | -0.5232 |
| Weighted-mean FA in tract forceps minor | -0.5113 |
| Mean FA in cerebral peduncle on FA skeleton (left) | -0.4964 |
| Volume of grey matter in Putamen (left) | 0.4557 |
| Mean L1 in middle cerebellar peduncle on FA skeleton | -0.4240 |
| Volume of grey matter in Insular Cortex (left) | 0.4107 |
| Volume of grey matter in VI Cerebellum (left) | -0.4004 |
| Volume of putamen (left) | -0.3795 |
| Median T2star in putamen (left) | -0.3773 |
| Volume of grey matter in IX Cerebellum (left) | 0.3740 |
| Mean L1 in anterior limb of internal capsule on FA skeleton (left) | 0.3556 |
| Mean MO in fornix cres+stria terminalis on FA skeleton (left) | -0.3367 |
| Weighted-mean OD in tract posterior thalamic radiation (right) | -0.3219 |
| Mean ICVF in superior longitudinal fasciculus on FA skeleton (right) | 0.3088 |
| Mean MO in fornix on FA skeleton | 0.3075 |
| Weighted-mean ISOVF in tract uncinate fasciculus (left) | 0.2999 |
| Volume of grey matter in Frontal Operculum Cortex (right) | -0.2969 |
| Mean L3 in posterior thalamic radiation on FA skeleton (right) | 0.2762 |
| Volume of thalamus (left) | -0.2757 |
| Mean L2 in fornix cres+stria terminalis on FA skeleton (left) | 0.2661 |
| Mean OD in posterior limb of internal capsule on FA skeleton (right) | 0.2650 |
| Mean FA in body of corpus callosum on FA skeleton | 0.2633 |
| Weighted-mean MO in tract acoustic radiation (left) | -0.2515 |
| Volume of grey matter in Heschl's Gyrus (includes H1 and H2) (right) | -0.2482 |
| Mean L1 in genu of corpus callosum on FA skeleton | 0.2473 |
| Mean L2 in splenium of corpus callosum on FA skeleton | -0.2430 |
| Weighted-mean ISOVF in tract forceps minor | -0.2387 |
| Volume of putamen (right) | -0.2383 |
| Mean OD in anterior limb of internal capsule on FA skeleton (right) | 0.2364 |
| Weighted-mean ISOVF in tract superior thalamic radiation (right) | 0.2271 |
| Volume of grey matter in Lateral Occipital Cortex, inferior division (left) | 0.2193 |
| Mean ICVF in body of corpus callosum on FA skeleton | 0.2128 |
| rfMRI partial correlation matrix, dimension 25^2^ (element 180) | 0.2090 |
| Mean ICVF in tapetum on FA skeleton (right) | -0.2087 |
| Median z-statistic (in group-defined amygdala activation mask) for faces-shapes contrast | -0.2082 |
| Mean OD in pontine crossing tract on FA skeleton | 0.2078 |
| Mean L3 in retrolenticular part of internal capsule on FA skeleton (left) | -0.2076 |
| Weighted-mean ISOVF in tract superior longitudinal fasciculus (right) | 0.2068 |
| Mean ISOVF in superior corona radiata on FA skeleton (right) | 0.1990 |
| rfMRI partial correlation matrix, dimension 25 (element 127) | 0.1965 |
| Mean L1 in sagittal stratum on FA skeleton (right) | -0.1930 |
| Mean ICVF in fornix cres+stria terminalis on FA skeleton (right) | -0.1921 |
| Weighted-mean L3 in tract medial lemniscus (right) | -0.1885 |
| rfMRI partial correlation matrix, dimension 25 (element 44) | -0.1877 |
| Volume of grey matter in Inferior Temporal Gyrus, temporooccipital part (left) | 0.1851 |
| Mean ICVF in retrolenticular part of internal capsule on FA skeleton (right) | 0.1833 |
| Mean FA in superior cerebellar peduncle on FA skeleton (left) | 0.1828 |
| rfMRI partial correlation matrix, dimension 25 (element 172) | 0.1826 |
| Weighted-mean L1 in tract parahippocampal part of cingulum (left) | -0.1823 |
| Weighted-mean ICVF in tract superior longitudinal fasciculus (right) | 0.1801 |
| Mean OD in superior cerebellar peduncle on FA skeleton (left) | -0.1790 |
| Volume of grey matter in Frontal Orbital Cortex (left) | -0.1696 |
| Volume of grey matter in X Cerebellum (left) | -0.1652 |
| Volume of grey matter in Paracingulate Gyrus (right) | -0.1651 |
| Weighted-mean L1 in tract corticospinal tract (left) | -0.1642 |
| Mean ICVF in medial lemniscus on FA skeleton (left) | 0.1599 |
| rfMRI partial correlation matrix, dimension 25 (element 22) | 0.1593 |
| Volume of grey matter in Hippocampus (left) | -0.1592 |
| Weighted-mean FA in tract middle cerebellar peduncle | -0.1569 |
| Mean ICVF in corticospinal tract on FA skeleton (right) | 0.1564 |
| 90th percentile of BOLD effect (in group-defined mask) for faces-shapes contrast | -0.1537 |
| Volume of grey matter in Crus II Cerebellum (vermis) | 0.1511 |
| rfMRI partial correlation matrix, dimension 25 (element 96) | 0.1498 |
| Weighted-mean ICVF in tract middle cerebellar peduncle | -0.1496 |
| Weighted-mean OD in tract anterior thalamic radiation (left) | -0.1485 |
| Mean L1 in superior corona radiata on FA skeleton (left) | 0.1481 |
| rfMRI partial correlation matrix, dimension 25 (element 151) | -0.1473 |
| rfMRI partial correlation matrix, dimension 25 (element 131) | -0.1440 |
| Mean L1 in superior longitudinal fasciculus on FA skeleton (left) | -0.1422 |
| Mean ISOVF in cingulum cingulate gyrus on FA skeleton (right) | -0.1402 |
| rfMRI partial correlation matrix, dimension 25 (element 89) | 0.1397 |
| Mean ICVF in corticospinal tract on FA skeleton (left) | 0.1388 |
| Volume of grey matter in Frontal Operculum Cortex (left) | -0.1377 |
| rfMRI partial correlation matrix, dimension 25 (element 46) | -0.1336 |
| Volume of grey matter in X Cerebellum (right) | -0.1322 |
| Mean FA in fornix on FA skeleton | -0.1321 |
| Median T2star in thalamus (left) | 0.1312 |
| Mean FA in corticospinal tract on FA skeleton (right) | 0.1299 |
| Weighted-mean MO in tract acoustic radiation (right) | -0.1284 |
| Volume of grey matter in Lingual Gyrus (right) | -0.1282 |
| Mean L1 in cerebral peduncle on FA skeleton (right) | -0.1270 |
| rfMRI partial correlation matrix, dimension 25 (element 146) | 0.1266 |
| rfMRI partial correlation matrix, dimension 25 (element 135) | -0.1256 |
| Weighted-mean L1 in tract corticospinal tract (right) | -0.1253 |
| Volume of grey matter in Angular Gyrus (left) | 0.1231 |
| Mean L2 in cingulum cingulate gyrus on FA skeleton (right) | -0.1207 |
| Volume of grey matter in Supramarginal Gyrus, anterior division (right) | 0.1195 |
| Volume of grey matter in Crus I Cerebellum (vermis) | -0.1187 |
| Volume of grey matter in Planum Polare (left) | -0.1185 |
| Volume of grey matter in Pallidum (left) | 0.1175 |
| Volume of grey matter in Planum Polare (right) | -0.1166 |
| Weighted-mean MO in tract forceps major | -0.1160 |
| Mean ICVF in posterior corona radiata on FA skeleton (left) | 0.1153 |
| Weighted-mean ICVF in tract anterior thalamic radiation (right) | -0.1150 |
| Volume of grey matter in Frontal Orbital Cortex (right) | -0.1143 |
| rfMRI partial correlation matrix, dimension 25 (element 159) | 0.1122 |
| rfMRI partial correlation matrix, dimension 25 (element 161) | -0.1119 |
| Weighted-mean OD in tract superior longitudinal fasciculus (left) | 0.1116 |
| Weighted-mean L2 in tract posterior thalamic radiation (right) | 0.1114 |
| rfMRI partial correlation matrix, dimension 25 (element 49) | -0.1111 |
| Weighted-mean ICVF in tract corticospinal tract (right) | 0.1107 |
| rfMRI partial correlation matrix, dimension 25 (element 202) | 0.1087 |
| Weighted-mean L1 in tract acoustic radiation (left) | -0.1083 |
| Mean MD in cingulum cingulate gyrus on FA skeleton (right) | -0.1082 |
| Volume of grey matter in Frontal Medial Cortex (right) | -0.1079 |
| Mean L1 in superior corona radiata on FA skeleton (right) | 0.1078 |
| rfMRI partial correlation matrix, dimension 25 (element 124) | -0.1065 |
| Mean FA in middle cerebellar peduncle on FA skeleton | -0.1061 |
| Volume of grey matter in Occipital Fusiform Gyrus (left) | 0.1054 |
| 90th percentile of z-statistic (in group-defined amygdala activation mask) for faces-shapes contrast | -0.1052 |
| rfMRI partial correlation matrix, dimension 25 (element 58) | 0.1025 |
| Mean ISOVF in inferior cerebellar peduncle on FA skeleton (left) | -0.1009 |
| Mean MO in medial lemniscus on FA skeleton (right) | 0.1004 |
| Mean FA in fornix cres+stria terminalis on FA skeleton (right) | -0.0995 |
| Volume of grey matter in Thalamus (right) | 0.0991 |
| Mean ICVF in posterior thalamic radiation on FA skeleton (left) | -0.0989 |
| Weighted-mean OD in tract posterior thalamic radiation (left) | -0.0978 |
| rfMRI partial correlation matrix, dimension 25 (element 66) | -0.0954 |
| rfMRI partial correlation matrix, dimension 25 (element 60) | -0.0949 |
| Mean OD in superior cerebellar peduncle on FA skeleton (right) | -0.0947 |
| Mean MO in medial lemniscus on FA skeleton (left) | 0.0945 |
| rfMRI partial correlation matrix, dimension 25 (element 155) | 0.0934 |
| rfMRI partial correlation matrix, dimension 25 (element 144) | -0.0924 |
| Weighted-mean MO in tract cingulate gyrus part of cingulum (left) | -0.0910 |
| Volume of grey matter in Inferior Temporal Gyrus, posterior division (right) | -0.0895 |
| rfMRI partial correlation matrix, dimension 25 (element 156) | 0.0885 |
| Weighted-mean MD in tract corticospinal tract (right) | -0.0885 |
| rfMRI partial correlation matrix, dimension 25 (element 130) | -0.0881 |
| Volume of grey matter in Crus II Cerebellum (right) | -0.0856 |
| Volume of grey matter in VIIIb Cerebellum (vermis) | 0.0850 |
| Median T2star in thalamus (right) | 0.0843 |
| Volume of grey matter in Supramarginal Gyrus, posterior division (right) | -0.0840 |
| rfMRI partial correlation matrix, dimension 25 (element 103) | -0.0840 |
| Mean MD in uncinate fasciculus on FA skeleton (right) | 0.0835 |
| Mean ICVF in superior fronto-occipital fasciculus on FA skeleton (right) | -0.0832 |
| rfMRI partial correlation matrix, dimension 25 (element 204) | 0.0819 |
| Weighted-mean ISOVF in tract middle cerebellar peduncle | -0.0816 |
| Mean MO in fornix cres+stria terminalis on FA skeleton (right) | -0.0807 |
| Mean L1 in superior fronto-occipital fasciculus on FA skeleton (left) | 0.0799 |
| Mean MO in external capsule on FA skeleton (right) | -0.0797 |
| Mean OD in fornix on FA skeleton | -0.0796 |
| rfMRI partial correlation matrix, dimension 25 (element 25) | -0.0790 |
| rfMRI partial correlation matrix, dimension 25 (element 121) | -0.0781 |
| Weighted-mean ISOVF in tract parahippocampal part of cingulum (right) | 0.0780 |
| rfMRI partial correlation matrix, dimension 25 (element 97) | 0.0779 |
| rfMRI partial correlation matrix, dimension 25 (element 125) | 0.0772 |
| Mean ICVF in inferior cerebellar peduncle on FA skeleton (left) | -0.0749 |
| Volume of grey matter in Planum Temporale (left) | -0.0745 |
| Mean FA in inferior cerebellar peduncle on FA skeleton (left) | -0.0728 |
| Weighted-mean MO in tract posterior thalamic radiation (left) | -0.0724 |
| Mean OD in posterior thalamic radiation on FA skeleton (right) | 0.0715 |
| rfMRI partial correlation matrix, dimension 25 (element 84) | -0.0707 |
| rfMRI partial correlation matrix, dimension 25 (element 55) | 0.0682 |
| rfMRI partial correlation matrix, dimension 25 (element 142) | -0.0678 |
| 90th percentile of BOLD effect (in group-defined amygdala activation mask) for faces-shapes contrast | 0.0666 |
| Mean OD in posterior limb of internal capsule on FA skeleton (left) | 0.0662 |
| Mean FA in anterior limb of internal capsule on FA skeleton (left) | 0.0661 |
| rfMRI partial correlation matrix, dimension 25 (element 101) | 0.0657 |
| rfMRI partial correlation matrix, dimension 25 (element 31) | 0.0647 |
| rfMRI partial correlation matrix, dimension 25 (element 205) | 0.0644 |
| rfMRI partial correlation matrix, dimension 25 (element 35) | -0.0634 |
| Weighted-mean L1 in tract superior thalamic radiation (right) | 0.0622 |
| Mean MD in retrolenticular part of internal capsule on FA skeleton (right) | -0.0617 |
| Weighted-mean MO in tract medial lemniscus (left) | -0.0614 |
| Mean OD in external capsule on FA skeleton (right) | 0.0612 |
| Mean ISOVF in posterior limb of internal capsule on FA skeleton (left) | -0.0589 |
| Median T2star in accumbens (left) | -0.0570 |
| Weighted-mean ISOVF in tract anterior thalamic radiation (left) | -0.0569 |
| rfMRI partial correlation matrix, dimension 25 (element 169) | 0.0560 |
| Mean MO in inferior cerebellar peduncle on FA skeleton (right) | -0.0556 |
| rfMRI partial correlation matrix, dimension 25 (element 92) | -0.0552 |
| Mean OD in cingulum cingulate gyrus on FA skeleton (left) | -0.0534 |
| rfMRI partial correlation matrix, dimension 25 (element 94) | 0.0528 |
| Mean OD in uncinate fasciculus on FA skeleton (right) | 0.0502 |
| Volume of grey matter in Cingulate Gyrus, anterior division (left) | -0.0490 |
| Weighted-mean OD in tract uncinate fasciculus (right) | -0.0490 |
| Weighted-mean OD in tract parahippocampal part of cingulum (right) | 0.0468 |
| Mean OD in anterior corona radiata on FA skeleton (right) | -0.0465 |
| Weighted-mean ISOVF in tract posterior thalamic radiation (right) | 0.0450 |
| rfMRI partial correlation matrix, dimension 25 (element 21) | -0.0448 |
| rfMRI partial correlation matrix, dimension 25 (element 194) | 0.0446 |
| Volume of grey matter in VI Cerebellum (right) | -0.0435 |
| rfMRI partial correlation matrix, dimension 25 (element 128) | 0.0433 |
| Mean ISOVF in cingulum hippocampus on FA skeleton (left) | 0.0425 |
| Mean OD in corticospinal tract on FA skeleton (left) | -0.0416 |
| rfMRI partial correlation matrix, dimension 25 (element 143) | 0.0412 |
| Mean MD in cingulum hippocampus on FA skeleton (right) | -0.0410 |
| rfMRI partial correlation matrix, dimension 25 (element 39) | -0.0407 |
| Volume of amygdala (left) | 0.0403 |
| rfMRI partial correlation matrix, dimension 25 (element 51) | -0.0399 |
| Mean L2 in posterior corona radiata on FA skeleton (left) | -0.0396 |
| rfMRI partial correlation matrix, dimension 25 (element 210) | 0.0390 |
| rfMRI partial correlation matrix, dimension 25 (element 95) | -0.0380 |
| Mean L2 in fornix cres+stria terminalis on FA skeleton (right) | 0.0377 |
| rfMRI partial correlation matrix, dimension 25 (element 50) | -0.0375 |
| rfMRI partial correlation matrix, dimension 25 (element 15) | 0.0372 |
| Weighted-mean MO in tract superior longitudinal fasciculus (right) | -0.0366 |
| Weighted-mean MD in tract superior thalamic radiation (left) | 0.0364 |
| Mean OD in cerebral peduncle on FA skeleton (right) | 0.0360 |
| Mean ISOVF in anterior limb of internal capsule on FA skeleton (right) | 0.0348 |
| Volume of grey matter in Subcallosal Cortex (right) | 0.0343 |
| Volume of grey matter in VIIb Cerebellum (vermis) | -0.0337 |
| Volume of grey matter in Occipital Pole (left) | -0.0329 |
| rfMRI partial correlation matrix, dimension 25 (element 189) | 0.0328 |
| Median T2star in pallidum (right) | 0.0326 |
| rfMRI partial correlation matrix, dimension 25 (element 183) | 0.0325 |
| Mean MD in cingulum cingulate gyrus on FA skeleton (left) | -0.0322 |
| Mean L1 in pontine crossing tract on FA skeleton | -0.0321 |
| rfMRI partial correlation matrix, dimension 25 (element 140) | -0.0305 |
| rfMRI partial correlation matrix, dimension 25 (element 75) | 0.0302 |
| rfMRI partial correlation matrix, dimension 25 (element 5) | 0.0301 |
| Volume of grey matter in Brain-Stem | -0.0301 |
| rfMRI partial correlation matrix, dimension 25 (element 176) | 0.0294 |
| rfMRI partial correlation matrix, dimension 25 (element 85) | -0.0294 |
| Mean L3 in posterior limb of internal capsule on FA skeleton (left) | -0.0290 |
| rfMRI partial correlation matrix, dimension 25 (element 82) | -0.0284 |
| rfMRI partial correlation matrix, dimension 25 (element 123) | -0.0283 |
| 90th percentile of BOLD effect (in group-defined mask) for shapes activation | 0.0279 |
| Volume of grey matter in Lingual Gyrus (left) | -0.0278 |
| rfMRI partial correlation matrix, dimension 25 (element 26) | 0.0269 |
| Mean ICVF in superior cerebellar peduncle on FA skeleton (right) | 0.0266 |
| Weighted-mean OD in tract inferior longitudinal fasciculus (right) | 0.0264 |
| rfMRI partial correlation matrix, dimension 25 (element 141) | 0.0259 |
| rfMRI partial correlation matrix, dimension 25 (element 106) | -0.0257 |
| rfMRI partial correlation matrix, dimension 25 (element 16) | 0.0245 |
| rfMRI partial correlation matrix, dimension 25 (element 112) | 0.0243 |
| Volume of grey matter in I-IV Cerebellum (right) | -0.0236 |
| rfMRI partial correlation matrix, dimension 25 (element 116) | -0.0235 |
| Weighted-mean ISOVF in tract anterior thalamic radiation (right) | -0.0232 |
| Median BOLD effect (in group-defined mask) for faces activation | 0.0229 |
| rfMRI partial correlation matrix, dimension 25 (element 64) | -0.0224 |
| Volume of grey matter in IX Cerebellum (right) | 0.0214 |
| Volume of grey matter in Temporal Fusiform Cortex, posterior division (left) | -0.0212 |
| rfMRI partial correlation matrix, dimension 25 (element 196) | -0.0201 |
| Weighted-mean MO in tract posterior thalamic radiation (right) | -0.0199 |
| rfMRI partial correlation matrix, dimension 25 (element 29) | 0.0190 |
| rfMRI partial correlation matrix, dimension 25 (element 71) | 0.0189 |
| Mean L2 in posterior limb of internal capsule on FA skeleton (right) | 0.0187 |
| rfMRI partial correlation matrix, dimension 25 (element 56) | -0.0180 |
| rfMRI partial correlation matrix, dimension 25 (element 17) | -0.0175 |
| Volume of grey matter in Inferior Temporal Gyrus, anterior division (right) | -0.0168 |
| Volume of grey matter in Juxtapositional Lobule Cortex (formerly Supplementary Motor Cortex) (left) | -0.0155 |
| Mean L2 in sagittal stratum on FA skeleton (left) | -0.0150 |
| rfMRI partial correlation matrix, dimension 25 (element 164) | 0.0149 |
| rfMRI partial correlation matrix, dimension 25 (element 133) | 0.0144 |
| Volume of grey matter in Lateral Occipital Cortex, superior division (right) | -0.0140 |
| rfMRI partial correlation matrix, dimension 25 (element 107) | 0.0136 |
| Volume of grey matter in Cuneal Cortex (right) | 0.0131 |
| Mean L1 in cerebral peduncle on FA skeleton (left) | -0.0126 |
| Volume of grey matter in Superior Temporal Gyrus, posterior division (left) | -0.0125 |
| Volume of grey matter in Supramarginal Gyrus, anterior division (left) | 0.0123 |
| rfMRI partial correlation matrix, dimension 25 (element 113) | -0.0123 |
| Mean L2 in retrolenticular part of internal capsule on FA skeleton (right) | -0.0121 |
| rfMRI partial correlation matrix, dimension 25 (element 80) | 0.0118 |
| Mean OD in retrolenticular part of internal capsule on FA skeleton (left) | 0.0109 |
| Mean MO in cerebral peduncle on FA skeleton (left) | -0.0108 |
| Volume of grey matter in Superior Parietal Lobule (left) | -0.0106 |
| rfMRI partial correlation matrix, dimension 25 (element 98) | 0.0094 |
| Weighted-mean OD in tract cingulate gyrus part of cingulum (left) | 0.0089 |
| Mean L1 in uncinate fasciculus on FA skeleton (right) | 0.0084 |
| rfMRI partial correlation matrix, dimension 25 (element 7) | -0.0081 |
| Weighted-mean OD in tract uncinate fasciculus (left) | 0.0079 |
| rfMRI partial correlation matrix, dimension 25 (element 197) | -0.0077 |
| Median T2star in pallidum (left) | 0.0067 |
| rfMRI partial correlation matrix, dimension 25 (element 190) | 0.0066 |
| rfMRI partial correlation matrix, dimension 25 (element 206) | 0.0063 |
| Weighted-mean MO in tract forceps minor | -0.0062 |
| Volume of grey matter in Frontal Pole (left) | -0.0060 |
| Mean L3 in posterior thalamic radiation on FA skeleton (left) | 0.0059 |
| rfMRI partial correlation matrix, dimension 25 (element 136) | -0.0057 |
| rfMRI partial correlation matrix, dimension 25 (element 179) | 0.0053 |
| Mean MO in cingulum hippocampus on FA skeleton (right) | 0.0044 |
| Mean OD in superior fronto-occipital fasciculus on FA skeleton (left) | -0.0034 |
| Weighted-mean OD in tract acoustic radiation (left) | 0.0023 |
| rfMRI partial correlation matrix, dimension 25 (element 186) | 0.0019 |
| Volume of grey matter in VI Cerebellum (vermis) | -0.0013 |
| rfMRI partial correlation matrix, dimension 25 (element 87) | -0.0008 |

^1^ The phenotypes with non-zero coefficient values have been selectively listed.

^2^ A partial correlation matrix with 25 dimensionalities (field ID: 25752), which had been converted into vectors with 210 elements.

# Supplementary Table 6. Standardized β coefficient and 95% confidence interval (CI) for the association of resting heart rate (RHR) with brain age and brain age gap (BAG): stratified by cardiovascular disease.

| **RHR** | **Brain age** | | **BAG** | |
| --- | --- | --- | --- | --- |
|  | β (95% CI) ^a^ | β (95% CI) ^b^ | β (95% CI) ^a^ | β (95% CI) ^b^ |
| **Non-cardiovascular disease (n = 18,259)** | | | | |
| Continuous (per 1-SD increase) | 0.364 (0.283, 0.446) ^†^ | 0.288 (0.196, 0.381) ^†^ | 0.322 (0.248, 0.396) ^†^ | 0.222 (0.138, 0.306) ^†^ |
| Categories |  |  |  |  |
| < 60 bpm | -0.405 (-0.607, -0.203) ^†^ | -0.320 (-0.542, -0.097) ^†^ | -0.362 (-0.546, -0.177) ^†^ | -0.245 (-0.448, -0.042) ^†^ |
| 60-69 bpm | Reference | Reference | Reference | Reference |
| 70-79 bpm | 0.197 (0.006, 0.389) | 0.193 (-0.018, 0.404) | 0.112 (-0.063, 0.286) | 0.069 (-0.124, 0.261) |
| ≥ 80 bpm | 0.799 (0.520, 1.077) ^†^ | 0.628 (0.315, 0.940) ^†^ | 0.768 (0.513, 1.023) ^†^ | 0.560 (0.275, 0.845) ^†^ |
| **Cardiovascular disease (n = 15,122)** | | | | |
| Continuous (per 1-SD increase) | 0.394 (0.312, 0.476) ^†^ | 0.376 (0.280, 0.471) ^†^ | 0.357 (0.281, 0.433) ^†^ | 0.307 (0.219, 0.396) |
| Categories |  |  |  |  |
| < 60 bpm | -0.283 (-0.538, -0.028) ^†^ | -0.336 (-0.623, -0.049) | -0.240 (-0.475, -0.005) | -0.218 (-0.482, 0.046) |
| 60-69 bpm | Reference | Reference | Reference | Reference |
| 70-79 bpm | 0.534 (0.299, 0.769) ^†^ | 0.564 (0.303, 0.825) ^†^ | 0.477 (0.260, 0.693) ^†^ | 0.484 (0.244, 0.725) ^†^ |
| ≥ 80 bpm | 1.022 (0.741, 1.304) ^†^ | 0.872 (0.556, 1.187) ^†^ | 0.944 (0.685, 1.203) ^†^ | 0.757 (0.466, 1.048) ^†^ |
| ***P_-interaction_*** | <0.001^a^ | 0.290 ^b^ | <0.001^a^ | 0.124 ^b^ |

^a^ Model adjusted for age, sex, and education.

^b^ Model adjusted for age, sex, education, race, Townsend deprivation index, body mass index, alcohol consumption, smoking, physical activity, social contact, diabetes, beta blockers, calcium blockers, and *APOE* ε4.

^†^ FDR *P* <0.05.

# Supplementary Table 7. Standardized β coefficient and 95% confidence interval (CI) for the association of resting heart rate (RHR) with brain age and brain age gap (BAG): stratified by *apolipoprotein E* epsilon 4 (*APOE* ε4).

| **RHR** | **Brain age** | | **BAG** | |
| --- | --- | --- | --- | --- |
|  | β (95% CI) ^a^ | β (95% CI) ^b^ | β (95% CI) ^a^ | β (95% CI) ^b^ |
| ***APOE* ε4 non-carriers (n = 20,215)** | | | | |
| Continuous (per 1-SD increase) | 0.431 (0.358, 0.504) ^†^ | 0.333 (0.256, 0.411) ^†^ | 0.386 (0.318, 0.453) ^†^ | 0.264 (0.193, 0.335) ^†^ |
| Categories |  |  |  |  |
| < 60 bpm | -0.279 (-0.482, -0.077) ^†^ | -0.301 (-0.508, -0.093) ^†^ | -0.224 (-0.410, -0.037) ^†^ | -0.215 (-0.405, -0.025) ^†^ |
| 60-69 bpm | Reference | Reference | Reference | Reference |
| 70-79 bpm | 0.547 (0.359, 0.736) ^†^ | 0.446 (0.254, 0.638) ^†^ | 0.431 (0.258, 0.605) ^†^ | 0.312 (0.136, 0.488) ^†^ |
| ≥ 80 bpm | 1.033 (0.785, 1.281) ^†^ | 0.686 (0.430, 0.942) ^†^ | 0.999 (0.771, 1.227) ^†^ | 0.611 (0.376, 0.845) ^†^ |
| ***APOE* ε4 carriers (n = 7,698)** | | | | |
| Continuous (per 1-SD increase) | 0.410 (0.286, 0.533) ^†^ | 0.334 (0.207, 0.462) ^†^ | 0.370 (0.256, 0.485) ^†^ | 0.268 (0.151, 0.385) ^†^ |
| Categories |  |  |  |  |
| < 60 bpm | -0.443 (-0.782, -0.104) ^†^ | -0.384 (-0.723, -0.044) | -0.353 (-0.667, -0.039) ^†^ | -0.266 (-0.578, 0.046) |
| 60-69 bpm | Reference | Reference | Reference | Reference |
| 70-79 bpm | 0.180 (-0.141, 0.502) | 0.144 (-0.176, 0.464) | 0.180 (-0.118, 0.478) | 0.115 (-0.179, 0.410) |
| ≥ 80 bpm | 1.148 (0.721, 1.574) ^†^ | 0.897 (0.469, 1.326) ^†^ | 1.045 (0.650, 1.440) ^†^ | 0.732 (0.338, 1.127) ^†^ |
| ***P_-interaction_*** | 0.219 ^b^ | | 0.537 ^b^ | |

^a^ Model adjusted for age, sex, and education.

^b^ Model adjusted for age, sex, education, race, Townsend deprivation index, body mass index, alcohol consumption, smoking, physical activity, social contact, hypertension, diabetes, beta blockers, and calcium blockers.

^†^ FDR *P* <0.05.

# Supplementary Table 8. Standardized β coefficient and 95% confidence interval (CI) for the association of resting heart rate (RHR) with brain age and brain age gap (BAG): stratified by physical activity.

| **RHR** | **Brain age** | | **BAG** | |
| --- | --- | --- | --- | --- |
|  | β (95% CI) ^a^ | β (95% CI) ^b^ | β (95% CI) ^a^ | β (95% CI) ^b^ |
| **Regular physical activity (n = 24,041)** | | | | |
| Continuous (per 1-SD increase) | 0.433 (0.365, 0.501) ^†^ | 0.357 (0.280, 0.433) ^†^ | 0.396 (0.334, 0.458) ^†^ | 0.278 (0.209, 0.348) ^†^ |
| Categories |  |  |  |  |
| < 60 bpm | -0.442 (-0.620, -0.264) ^†^ | -0.378 (-0.572, -0.183) ^†^ | -0.382 (-0.545, -0.219) ^†^ | -0.282 (-0.460, -0.104) ^†^ |
| 60-69 bpm | Reference | Reference | Reference | Reference |
| 70-79 bpm | 0.362 (0.184, 0.539) ^†^ | 0.368 (0.175, 0.560) ^†^ | 0.305 (0.143, 0.468) ^†^ | 0.252 (0.076, 0.428) ^†^ |
| ≥ 80 bpm | 1.003 (0.762, 1.244) ^†^ | 0.744 (0.478, 1.011) ^†^ | 0.975 (0.754, 1.196) ^†^ | 0.637 (0.394, 0.880) ^†^ |
| **Unregular physical activity (n = 8,239)** | | | | |
| Continuous (per 1-SD increase) | 0.358 (0.240, 0.476) ^†^ | 0.239 (0.105, 0.372) ^†^ | 0.338 (0.229, 0.448) ^†^ | 0.199 (0.075, 0.323) ^†^ |
| Categories |  |  |  |  |
| < 60 bpm | 0.002 (-0.383, 0.387) | 0.003 (-0.425, 0.431) | 0.013 (-0.344, 0.370) | 0.078 (-0.318, 0.475) |
| 60-69 bpm | Reference | Reference | Reference | Reference |
| 70-79 bpm | 0.428 (0.134, 0.723) ^†^ | 0.326 (0.007, 0.646) | 0.345 (0.072, 0.618) ^†^ | 0.246 (-0.050, 0.541) |
| ≥ 80 bpm | 1.024 (0.666, 1.382) ^†^ | 0.717 (0.322, 1.113) ^†^ | 0.988 (0.656, 1.320) ^†^ | 0.634 (0.268, 1.000) ^†^ |
| ***P_-interaction_*** | 0.447 ^b^ | | 0.422 ^b^ | |

^a^ Model adjusted for age, sex, and education.

^b^ Model adjusted for age, sex, education, race, Townsend deprivation index, body mass index, alcohol consumption, smoking, social contact, hypertension, diabetes, beta blockers, calcium blockers, and *APOE* ε4.

^†^ FDR *P* <0.05.

# Supplementary Table 9. Standardized β coefficient and 95% confidence interval (CI) for the association of resting heart rate (RHR) with brain age and brain age gap (BAG): stratified by PRS_AD_.

| **RHR** | **Brain age** | | **BAG** | |
| --- | --- | --- | --- | --- |
|  | β (95% CI) ^a^ | β (95% CI) ^b^ | β (95% CI) ^a^ | β (95% CI) ^b^ |
| ***Low genetic risk* (n = 10,833)** | | | | |
| Continuous (per 1-SD increase) | 0.458 (0.358, 0.557) ^†^ | 0.354 (0.238, 0.470) ^†^ | 0.434 (0.342, 0.525) ^†^ | 0.288 (0.182, 0.395) ^†^ |
| Categories |  |  |  |  |
| < 60 bpm | -0.433 (-0.713, -0.153) ^†^ | -0.379 (-0.687, -0.070) ^†^ | -0.384 (-0.642, -0.127) ^†^ | -0.279 (-0.562, 0.003) |
| 60-69 bpm | **Reference** | **Reference** | **Reference** | **Reference** |
| 70-79 bpm | 0.446 (0.185, 0.707) ^†^ | 0.462 (0.175, 0.749) ^†^ | 0.377 (0.137, 0.617) ^†^ | 0.345 (0.082, 0.608) ^†^ |
| ≥ 80 bpm | 0.950 (0.613, 1.288) ^†^ | 0.548 (0.166, 0.929) ^†^ | 0.952 (0.641, 1.262) ^†^ | 0.489 (0.140, 0.839)^3^ |
| ***Moderate genetic risk* (n = 10,833)** | | | | |
| Continuous (per 1-SD increase) | 0.405 (0.303, 0.506) ^†^ | 0.324 (0.208, 0.439) ^†^ | 0.387 (0.293, 0.481) ^†^ | 0.278 (0.171, 0.384) ^†^ |
| Categories |  |  |  |  |
| < 60 bpm | -0.376 (-0.662, -0.090) ^†^ | -0.389 (-0.701, -0.077) ^†^ | -0.357 (-0.621, -0.092) ^†^ | -0.301 (-0.589, -0.013) |
| 60-69 bpm | **Reference** | **Reference** | **Reference** | **Reference** |
| 70-79 bpm | 0.418 (0.153, 0.683) ^†^ | 0.290 (0.001, 0.579) | 0.350 (0.105, 0.596) ^†^ | 0.192 (-0.075, 0.459) |
| ≥ 80 bpm | 0.969 (0.624, 1.313) ^†^ | 0.742 (0.361, 1.122) ^†^ | 0.999 (0.680, 1.318) ^†^ | 0.728 (0.376, 1.079) ^†^ |
| ***High genetic risk* (n = 10,832)** | | | | |
| Continuous (per 1-SD increase) | 0.434 (0.334, 0.534) ^†^ | 0.317 (0.203, 0.431) ^†^ | 0.375 (0.283, 0.466) ^†^ | 0.224 (0.120, 0.328) ^†^ |
| Categories |  |  |  |  |
| < 60 bpm | -0.242 (-0.516, 0.032) | -0.215 (-0.516, 0.086) | -0.183 (-0.433, 0.068) | -0.123 (-0.397, 0.152) |
| 60-69 bpm | **Reference** | **Reference** | **Reference** | **Reference** |
| 70-79 bpm | 0.376 (0.119, 0.634) ^†^ | 0.334 (0.053, 0.616) ^†^ | 0.299 (0.064, 0.534) ^†^ | 0.227 (-0.029, 0.484) |
| ≥ 80 bpm | 1.321 (0.976, 1.666) ^†^ | 0.923 (0.540, 1.306) ^†^ | 1.173 (0.857, 1.488) ^†^ | 0.697 (0.347, 1.046) ^†^ |
| ***P_-interaction_*** | 0.137 ^b^ | | 0.224 ^b^ | |

^a^ Model adjusted for age, sex, and education.

^b^ Model adjusted for age, sex, education, race, Townsend deprivation index, body mass index, alcohol consumption, smoking, physical activity, social contact, hypertension, diabetes, beta blockers, and calcium blockers.

^†^ FDR *P* <0.05.

# Supplementary Table 10. Standardized β coefficient and 95% confidence interval (CI) for the association of resting heart rate (RHR) with brain age and brain age gap (BAG): further adjusted for DASH diet score.

| **RHR** | **Brain Age** | **BAG** |
| --- | --- | --- |
|  | **β (95% CI) ^a^** | **β (95% CI) ^a^** |
| **All participants** | | |
| Continuous | 0.355 (0.253, 0.457) ^†^ | 0.288 (0.194, 0.381) ^†^ |
| Categorical |  |  |
| <60 bpm | -0.408 (-0.684, -0.133) ^†^ | -0.288 (-0.540, -0.035) ^†^ |
| 60-69 bpm | **Reference** | **Reference** |
| 70-79 bpm | 0.473 (0.216, 0.731) ^†^ | 0.388 (0.152, 0.624) ^†^ |
| ≥80 bpm | 0.793 (0.452, 1.135) ^†^ | 0.691 (0.378, 1.004) ^†^ |
| **Middle-aged (40-60 years)** | | |
| Continuous | 0.233 (0.114, 0.351) ^†^ | 0.165 (0.056, 0.274) ^†^ |
| Categorical |  |  |
| <60 bpm | -0.349 (-0.666, -0.031) | -0.259 (-0.551, 0.033) |
| 60-69 bpm | **Reference** | **Reference** |
| 70-79 bpm | 0.323 (0.029, 0.618) | 0.235 (-0.036, 0.506) |
| ≥80 bpm | 0.425 (0.031, 0.818) | 0.287 (-0.074, 0.649) |
| **Older-aged (60+ years)** | | |
| Continuous | 0.584 (0.387, 0.781) ^†^ | 0.521 (0.342, 0.700) ^†^ |
| Categorical |  |  |
| <60 bpm | -0.547 (-1.084, -0.010) | -0.353 (-0.841, 0.136) |
| 60-69 bpm | **Reference** | **Reference** |
| 70-79 bpm | 0.768 (0.255, 1.281) ^†^ | 0.691 (0.224, 1.157) ^†^ |
| ≥80 bpm | 1.511 (0.843, 2.180) ^†^ | 1.504 (0.896, 2.112) ^†^ |

Abbreviation: CI, confidence interval.

^a^ Model adjusted for age, sex, education, race, Townsend deprivation index, body mass index, alcohol consumption, smoking, physical activity, social contact, hypertension, diabetes, beta blockers, calcium blockers, *APOE* ε4, and DASH diet score.

^†^ FDR *P* <0.05.

# Supplementary Table 11. Standardized β coefficient and 95% confidence interval (CI) for the association of resting heart rate (RHR) with brain age and brain age gap (BAG): further adjusted for PRS_AD_.

| **RHR** | **Brain Age** | **BAG** |
| --- | --- | --- |
|  | **β (95% CI) ^a^** | **β (95% CI) ^a^** |
| **All participants** | | |
| Continuous | 0.355 (0.253, 0.457) ^†^ | 0.288 (0.194, 0.381) ^†^ |
| Categorical |  |  |
| <60 bpm | -0.408 (-0.684, -0.133) ^†^ | -0.288 (-0.540, -0.035) ^†^ |
| 60-69 bpm | **Reference** | **Reference** |
| 70-79 bpm | 0.473 (0.216, 0.731) ^†^ | 0.388 (0.152, 0.624) ^†^ |
| ≥80 bpm | 0.793 (0.452, 1.135) ^†^ | 0.691 (0.378, 1.004) ^†^ |
| **Middle-aged (40-60 years)** | | |
| Continuous | 0.233 (0.114, 0.351) ^†^ | 0.165 (0.056, 0.274) ^†^ |
| Categorical | **Reference** | **Reference** |
| <60 bpm | -0.349 (-0.666, -0.031) | -0.259 (-0.551, 0.033) |
| 60-69 bpm |  |  |
| 70-79 bpm | 0.323 (0.029, 0.618) | 0.235 (-0.036, 0.506) |
| ≥80 bpm | 0.425 (0.031, 0.818) | 0.287 (-0.074, 0.649) |
| **Older-aged (60+ years)** | | |
| Continuous | 0.584 (0.387, 0.781) ^†^ | 0.521 (0.342, 0.700) ^†^ |
| Categorical |  |  |
| <60 bpm | -0.547 (-1.084, -0.010) | -0.353 (-0.841, 0.136) |
| 60-69 bpm | **Reference** | **Reference** |
| 70-79 bpm | 0.768 (0.255, 1.281) ^†^ | 0.691 (0.224, 1.157) ^†^ |
| ≥80 bpm | 1.511 (0.843, 2.180) ^†^ | 1.504 (0.896, 2.112) ^†^ |

Abbreviation: CI, confidence interval.

^a^ Model adjusted for age, sex, education, race, Townsend deprivation index, body mass index, alcohol consumption, smoking, physical activity, social contact, hypertension, diabetes, beta blockers, calcium blockers, *APOE* ε4, and PRS_AD_.

^†^ FDR *P* <0.05.

# Supplementary Table 12. Standardized β coefficient and 95% confidence interval (CI) for the association of resting heart rate (RHR) with brain age and brain age gap (BAG): after multiple imputation of covariates.

| **RHR** | **Brain Age** | **BAG** |
| --- | --- | --- |
|  | **β (95% CI) ^a^** | **β (95% CI) ^a^** |
| **All participants** | | |
| Continuous | 0.358 (0.290, 0.426) ^†^ | 0.285 (0.223, 0.348) ^†^ |
| Categorical |  |  |
| <60 bpm | -0.325 (-0.500, -0.151) ^†^ | -0.231 (-0.391, -0.071) ^†^ |
| 60-69 bpm | **Reference** | **Reference** |
| 70-79 bpm | 0.694 (0.503, 0.884) ^†^ | 0.261 (0.112, 0.409) ^†^ |
| ≥80 bpm | 1.105 (0.866, 1.345) ^†^ | 0.670 (0.473, 0.808) ^†^ |
| **Middle-aged (40-60 years)** | | |
| Continuous | 0.294 (0.215, 0.374) ^†^ | 0.226 (0.152, 0.300 ^†^ |
| Categorical |  |  |
| <60 bpm | -0.224 (-0.048, 0.495) | -0.223 (-0.411, 0.035) |
| 60-69 bpm | **Reference** | **Reference** |
| 70-79 bpm | 0.264 (-0.033, 0.560) | 0.262 (0.088, 0.435) |
| ≥80 bpm | 0.375 (0.001, 0.750) | 0.545 (0.312, 0.778) ^†^ |
| **Older-aged (60+ years)** | | |
| Continuous | 0.466 (0.337, 0.595) ^†^ | 0.387 (0.270, 0.504) ^†^ |
| Categorical |  |  |
| <60 bpm | 0.507 (-0.139, 0.874) | -0.162 (-0.566, 0.42) |
| 60-69 bpm | **Reference** | **Reference** |
| 70-79 bpm | 0.967 (0.565, 1.368) ^†^ | 0.290 (0.005, 0.575) ^†^ |
| ≥80 bpm | 1.534 (1.036, 2.033) ^†^ | 1.012 (0.638, 1.385) ^†^ |

Abbreviation: CI, confidence interval.

^a^ Model adjusted for age, sex, education, race, Townsend deprivation index, body mass index, alcohol consumption, smoking, physical activity, social contact, hypertension, diabetes, beta blockers, calcium blockers, and *APOE* ε4.

^†^ FDR *P* <0.05.
